# Association of variants in the *KIF1A* gene with amyotrophic lateral sclerosis

**DOI:** 10.1186/s40035-022-00320-2

**Published:** 2022-10-26

**Authors:** Panlin Liao, Yanchun Yuan, Zhen Liu, Xiaorong Hou, Wanzhen Li, Jin Wen, Kexuan Zhang, Bin Jiao, Lu Shen, Hong Jiang, Jifeng Guo, Beisha Tang, Zhuohua Zhang, Zhonghua Hu, Junling Wang

**Affiliations:** 1 Hunan Key Laboratory of Molecular Precision Medicine, Department of Critical Care Medicine, Xiangya Hospital, Central South University, Changsha, 410008 China; 2 Department of Neurology, Xiangya Hospital, Central South University, Changsha, 410008 China; 3 National Clinical Research Center for Geriatric Diseases, Xiangya Hospital, Central South University, Changsha, 410008 China; 4 Key Laboratory of Hunan Province in Neurodegenerative Disorders, Central South University, Changsha, 410008 China; 5 Center for Medical Genetics, School of Life Sciences, Central South University, Changsha, 410008 China; 6 Engineering Research Center of Hunan Province in Cognitive Impairment Disorders, Central South University, Changsha, 410008 China; 7 Hunan International Scientific and Technological Cooperation Base of Neurodegenerative and Neurogenetic Diseases, Changsha, 410008 China; 8 Hunan Provincial Clinical Research Center for Critical Care Medicine, Xiangya Hospital, Central South University, Changsha, 410008 China; 9 Hunan Key Laboratory of Animal Models for Human Diseases, School of Life Sciences, Central South University, Changsha, 410008 China

**Keywords:** Amyotrophic lateral sclerosis, KIF1A, Axonal transport, Presynaptic vesicle precursors

## Abstract

**Background:**

Amyotrophic lateral sclerosis (ALS) is a devastating progressive neurodegenerative disease that affects neurons in the central nervous system and the spinal cord. As in many other neurodegenerative disorders, the genetic risk factors and pathogenesis of ALS involve dysregulation of cytoskeleton and neuronal transport. Notably, sensory and motor neuron diseases such as hereditary sensory and autonomic neuropathy type 2 (HSAN2) and spastic paraplegia 30 (SPG30) share several causative genes with ALS, as well as having common clinical phenotypes. *KIF1A* encodes a kinesin 3 motor that transports presynaptic vesicle precursors (SVPs) and dense core vesicles and has been reported as a causative gene for HSAN2 and SPG30.

**Methods:**

Here, we analyzed whole-exome sequencing data from 941 patients with ALS to investigate the genetic association of *KIF1A* with ALS.

**Results:**

We identified rare damage variants (RDVs) in the *KIF1A* gene associated with ALS and delineated the clinical characteristics of ALS patients with *KIF1A* RDVs. Clinically, these patients tended to exhibit sensory disturbance. Interestingly, the majority of these variants are located at the C-terminal cargo-binding region of the KIF1A protein. Functional examination revealed that the ALS-associated *KIF1A* variants located in the C-terminal region preferentially enhanced the binding of SVPs containing RAB3A, VAMP2, and synaptophysin. Expression of several disease-related *KIF1A* mutants in cultured mouse cortical neurons led to enhanced colocalization of RAB3A or VAMP2 with the KIF1A motor.

**Conclusions:**

Our study highlighted the importance of KIF1A motor-mediated transport in the pathogenesis of ALS, indicating *KIF1A* as an important player in the oligogenic scenario of ALS.

**Supplementary Information:**

The online version contains supplementary material available at 10.1186/s40035-022-00320-2.

## Background

Amyotrophic lateral sclerosis (ALS) is a devastating neurodegenerative disease characterized by progressive degeneration of the upper and lower motor neurons, leading to muscular weakness and atrophy [1, 2]. ALS typically occurs in individuals between ages 42 and 65 and invariably leads to death due to respiratory failure three to four years after disease onset [1, 2]. Growing evidence shows that genetic risk factors, environmental exposure, and aging are implicated in the pathogenesis of ALS [3, 4]. Approximately 10% of ALS cases are familial ALS (FALS), and the remaining cases are classified as sporadic ALS (SALS). In recent years, with the advent of next-generation sequencing technology, more than 40 genes have been discovered to be involved in the pathogenesis of ALS [3, 5].

Despite the increase in genetic insights and the characterization of numerous ALS-associated genes, the biological mechanisms underlying how ALS genes contribute to the ALS phenotype remain to be elucidated. Various lines of genetic, pathological and neurobiological evidence have identified axon transport deficits as essential pathogenic mechanisms for ALS [6]. First, many ALS-associated genes (such as *PFN1*, *TUBA4A*, *DCTN1*, *ALS2*, *NEFH*, *KIF5A*, and *SPAST*) are known to regulate cytoskeletal dynamics and function as well as intracellular transport events [3, 6–9]. Second, animal studies have shown that defects in axonal transport and distal axonal damage are key pathogenic features in ALS animal models and precede the ALS-like symptoms in these animals [10–15]. Third, neuropathological analysis has indicated that some ALS patients exhibit denervation and reinnervation changes in muscles but normal-appearing motor neurons, suggesting that axonal damage occurs earlier than the initial apparent symptoms in ALS, that is, before clinical manifestations of disease [16]. Moreover, axonal cytoskeletal disorganization and defective transportation have been reported in a series of other neurodegenerative diseases, such as spastic paraplegia (SPG), Charcot-Marie-Tooth disease, and Parkinson’s disease [17, 18], highlighting axonal transport as a potential convergent mechanism of pathological neurodegeneration.

*KIF1A* encodes a kinesin-3 molecular motor [19] that transports both synaptic vesicle precursors (SVPs) carrying synaptic vesicle proteins (such as synaptophysin, VAMP2, and RAB3A) [20–22] and dense core vesicles (DCVs) carrying neuropeptides and neurotrophic factors [23–25]. *KIF1A* deficiency in mice leads to remarkable neuronal degeneration and death [26], indicating a pathogenic association with neurodegenerative conditions. Moreover, *KIF1A* heterozygous mutations have been linked to a variety of neurodegenerative and neurodevelopmental diseases [27]. Currently, mutations in *KIF1A* are listed as associated with three disorders in the OMIM database: hereditary sensory and autonomic neuropathy type 2 (HSAN2) with a recessive pattern; mental retardation type 9 (MRD9) with dominant inheritance; and SPG30 with either dominant or recessive inheritance. HSAN and SPG are known to share pathogenic genes with ALS. For example, *KIF5A* and *SPG11* are characterized as common causative genes of both ALS and SPG [28–31], and the *SPTLC1* gene, which causes HSAN, has recently been identified as a causative gene of juvenile ALS [32, 33]. In addition, SPG overlaps with ALS in terms of clinical symptoms and genetic risk factors. Thus, we hypothesize that the *KIF1A*-related mutations are a genetic risk of ALS pathogenesis.

In this study, we performed whole-exome sequencing (WES) in 941 patients with ALS, validated the candidate variants in an additional publicly available cohort of 4366 patients with ALS. We identified rare damage variants (RDVs) in the *KIF1A* gene associated with ALS, and investigated the functional effects of these variants on axonal transport.

## Methods

### Patients and clinical analysis

In this study, 941 patients with ALS, including 55 FALS and 886 SALS patients, were enrolled for mutation screening of candidate genes. This ALS cohort included 753 ALS patients whom we had reported on previously [2]. Among these patients, pathogenic nucleotide repeat expansion mutations in *C9ORF72* and *ATXN2* were excluded by PCR and repeat-primer PCR analysis. A comprehensive battery of clinical data including age, sex, family history and clinical features such as age at onset (AAO), site of onset, disease duration, and ALS Functional Rating Scale–Revised score were collected from all participants. To perform burden analysis, we selected WES data from 6708 East Asian individuals without any neurological disease in the gnomAD database v2 as the control for our ALS Chinese cohort. In addition, burden analysis was also performed in the Project MinE cohort (including 4366 ALS patients and 1832 controls) [34, 35]. In addition, *KIF1A* variants were screened in an independent ALS cohort from a publicly available dataset (ALSdb, New York City, New York (URL: http://alsdb.org) [Mar, 2022]), which consists of 3317 SALS patients who have undergone WES [36].

### WES analysis

All patients with ALS underwent WES using a previously described method [2, 37]. Variants that failed to meet our quality control requirements (coverage depth < 10, allele balance < 0.25, and Phred quality score < 20) were excluded. RDV was included for further analysis if it was (1) located in the *KIF1A* gene; (2) rare, i.e., for heterozygous variants, a minor allele frequency (MRD) less than 0.1% in the 1000 Genomes Project, Exome Aggregation Consortium (ExAC) and gnomAD; for homozygous or compound heterozygous variants, a minor allele frequency (MAF) less than 1% in the above public databases; (3) a nonsynonymous, indel, or putative splice site variant; and (4) pathogenic. For SALS, the pathogenicity was predicted by ReVe, a pathogenicity-computation method [38], with an ReVe value more than 0.7; for FALS, the pathogenicity was identified based on co-segregation results and prediction of ReVe. Then, the variants identified by WES were validated by Sanger sequencing.

### Plasmids and reagents

To construct the pGW1-KIF1A-3xFLAG plasmid, *KIF1A* cDNA was obtained by RT-PCR from mRNA extracted from human embryonic stem cells and cloned into the pGW1 vector [39] between the EcoRI and HindIII sites. The FLAG tag sequence was added to the reverse primer during PCR. To generate the pGW1-KIF1A-Venus or the pGW1-RFP-RAB3A construct, the cDNAs of KIF1A and RAB3A were obtained by RT-PCR and cloned into the pGW1 vector between the EcoRI and HindIII sites by using the Gibson assembly method. The pCMV-3xFLAG-RAB3A plasmid was constructed by PCR amplifying RAB3A from pGW1-RFP-RAB3A and subcloning the PCR fragment into the 3xFLAG-pCMV vector (Sigma, St. Louis, MO; E7658) between the EcoRI and BamHI sites. KIF1A point variants were designed using the NEBase Changer website. All clones were sequenced, and the expression of correctly sized proteins was confirmed by Western blot. FLAG-VAMP2 and RFP-synaptophysin plasmids were as described previously [40, 41]. The primer sequences used for constructing these plasmids are listed in Additional file 1: Table S1.

The following antibodies were used: DYKDDDDK Tag (CST, Danvers, MA; 8146), GFP (Invitrogen, Waltham, MA; A11122), actin (CST, 4970S), tubulin (TransGen, Beijing, China, P10513), GAPDH (Abcam, Cambridge, United Kingdom, ab8245), RFP (Rockland, Limerick, PA; 600-401-379), Alexa Fluor 555 donkey anti-mouse IgG (H + L) (Invitrogen, A21422), goat anti-rabbit IgG-HRP (Absin, Shanghai, China, abs20040ss), and goat anti-mouse IgG-HRP (Absin, abs20039ss).

### Coimmunoprecipitation

HEK293T cells were cultured in DMEM (Gibco, Waltham, MA; 11995500) supplemented with 10% fetal bovine serum (ProCell, Wuhan, China, 164210-50) and 100 U/ml penicillin–streptomycin solution (Thermo Fisher, Waltham, MA; 15140148). The cells were transfected using a GenJet instrument (SignaGen, Frederick, MD; SL100488) according to the manufacturer’s instructions. Forty-eight hours after transfection, cells were lysed in coimmunoprecipitation buffer (20 mM Tris-HCl pH 7.5, 150 mM NaCl, 1 mM EGTA, 1% Triton X-100 and proteinase inhibitors) at 4 °C for 30 min and then centrifuged at 13,000 rpm at 4 °C for 30 min to separate the soluble and insoluble fractions. Approximately 10%–20% of the supernatant was used as the input control, and the remainder was used for immunoprecipitation. The input was mixed with 2 × Laemmli sample buffer (60 mM Tris-HCl pH 6.8, 2% SDS, 10% glycerol, 5% β-mercaptoethanol, 0.01% bromophenol blue) and boiled at 95 °C for 10 min. The remainder of the supernatant was incubated with anti-FLAG M2 beads (Sigma, A2220) for 4 h at 4 °C. The beads were washed with coimmunoprecipitation buffer three times, and then the protein complexes were eluted with 2% SDS.

### Western blot

Cell lysates or immunoprecipitated proteins were separated by SDS-PAGE and then transferred to nitrocellulose filter membranes. After blocking with 5% nonfat milk in TBST (10 mM Tris, 70 mM NaCl, pH 7.6, 0.1% Tween-20), the membranes were incubated with primary antibodies overnight at 4 °C, washed with TBST three times for 15 min each, and then incubated with the corresponding HRP-conjugated secondary antibodies for one hour at room temperature. Quantification of blots was performed using ImageJ software (NIH, Bethesda, MA).

### Neuronal culture and transfection

Primary mouse cortical neuron cultures were prepared from E17.5 C57/B6L embryos as previously described [40]. Cortical tissues were dissected from embryos in precooled Hanks' Balanced Salt Solution (HBSS) and digested in prewarmed HBSS containing 0.025% trypsin (Gibco, 15090-046) at 37 °C for 20 min. Neurons were plated on poly-*D*-lysine (Sigma, P1024)-coated coverslips (200,000 cells/ml for 12-well plates, 500,000 cells/ml for 6-well plates). The cultures were maintained at 37 °C in 5% CO_2_ in neurobasal medium (Gibco, 21103049) containing 1 × B27 (Gibco, 17504044), 1 × GlutaMAX (Thermo Fisher, 35050061), and 100 U/ml penicillin–streptomycin solution (Thermo Fisher, 15140148). After seven days in vitro (DIV), the cultured neurons were transfected with plasmids using Lipofectamine 2000 Transfection Reagent (Thermo Fisher, 11668019).

### Immunofluorescence and image analysis

At four days after transfection, the cultured neurons were fixed with 4% paraformaldehyde (Polysciences, Warrington, PA; 04018-1) and 4% sucrose in PBS for 15 min at room temperature, followed by three washes with PBS. The cells were then incubated with primary antibody overnight at 4 °C, washed three times in PBS, and then incubated with secondary antibodies for 1 h at room temperature. Subsequently, the cells were washed three times in PBS and mounted in VECTASHIELD Mounting Media (Vector Labs, Newark, CA; H-1000). Z-stack images were acquired with a Zeiss LSM880 confocal microscope. Quantification of colocalized RAB3A/KIF1A or VAMP2/KIF1A was performed with Imaris software (Oxford Instruments, Abingdon, United Kingdom).

### Statistical analysis

Statistical analysis was performed with SPSS 25.0. Descriptive statistics (mean ± SD) were calculated for continuous variables. One-way ANOVA and Dunn’s test were used to compare continuous variable data. For comparison of categorical variables, Fisher’s exact test was applied. Association analysis of the RDVs was performed across the entire *KIF1A* gene and certain regions using Fisher's exact test, which was performed on an allelic basis. A *P* level of 0.05 was defined as the threshold of statistical significance.

## Results

### Mutation analysis of *KIF1A* in the ALS cohorts

To identify the possible association of *KIF1A* with ALS, we analyzed the WES data from 941 patients with ALS. Demographic and clinical characteristics are shown in Table 1. In this cohort, eight different candidate heterozygous RDVs in the *KIF1A* gene were identified in 10 unrelated patients, accounting for 1.06% of all patients (10/941). Among the 10 patients, two patients had family history of ALS, and the remaining patients were sporadic. The detailed information is listed in Additional file 1: Tables S2 and S3. Some RDVs deserve noticeable. The variant c.4760C > T; p.P1587L was identified in three unrelated sporadic patients (A737, A893, and A342). Notably, the c.4928C > T; p.A1643V variant was not found in any public database. We were unable to determine whether these variants were de novo variants, as samples from these patients’ parents could not be obtained because the parents had died or were otherwise unavailable. In addition, the patient P1 (A148) who had the c.1108C > T; p.R370C variant also carried a stop-gain variant in the *CHCHD10* gene (NM_213720:c.C312G; p.Y104X).

**Table 1:** Summary of demographic and clinical characteristics of ALS patients

	ALS cohort
No of ALS patients	941
Family history (+ ,%)	55, 5.84%
Sex (male, %)	616, 65.5%
Age at onset (years)	54.18 ± 10.83

In the Project Mine, 18 different rare nonsynonymous variants in *KIF1A* were identified in 19 ALS patients, including 17 missense variants and one frameshift variant. In the ALSdb cohort, additional 15 different rare nonsynonymous variants in *KIF1A* were identified in 16 ALS patients, including 14 missense variants and one stop-gain variant. In total, 45 patients with different RDVs in the *KIF1A* coding sequence were found among 8624 patients with ALS. All missense variants were predicted to be deleterious by at least six of 11 in silico tools and had an MAF < 0.1% in the public databases. The details of these variants and pathogenicity predictions are listed in Additional file 1: Tables S2 and S3.

### Burden analysis at the KIF1A gene level and C-terminal region level

A total of 10 putative pathogenic variants were identified in 941 patients with ALS. Among the WES data for the 6708 East Asian control individuals in the gnomAD database v2, we identified 33 RDVs in *KIF1A* that fulfilled the same screening criteria (Additional file 1: Tables S2 and S3, Fig. S1). At the level of the entire *KIF1A* gene, gene burden testing was significant for *KIF1A* variants as a risk factor for ALS (Fisher's exact test, *P* = 0.036, odd ratio [OR] = 2.17, 95% confidence interval [CI] = 1.07 to 4.40). The KIF1A protein is composed of an N-terminal motor domain, four coiled coil (CC1-CC4) domains, with a Forkhead associated domain (FHA) following the second CC domain, and a C-terminal pleckstrin homology domain (PH) (https://www.uniprot.org/). Interestingly, we found that the variants in *KIF1A* associated with the ALS phenotype were mainly located in the C-terminal cargo-binding region. We therefore selected variants located in the C-terminal cargo-binding region (exons 24–47, amino acids 747–1690, with reference to transcript NM_004321) for further statistical analysis. Finally, in the C-terminal region, we discovered nine variants in ALS patients and 18 variants in the East Asian control individuals without neurological disease in the gnomAD database v2. There was a significant association between variants in C-terminal regions and the ALS phenotype (Fisher's exact test, *P* = 0.004, OR = 3.58, 95%CI = 1.60–7.98). In the project Mine ALS cohort, burden analysis at the entire gene level and the C-terminal region level did not reach significant level (Table 2).

**Table 2:** Results of burden analysis at the *KIF1A* gene level in different cohorts

	Entire gene				C-terminal cargo-binding region			
Cohort	AC (patients)	AC (controls)	*P* value	OR [95% CI]	AC (patients)	AC (controls)	*P* value	OR [95% CI]
This study	10 (941)	33 (6708)	0.036*	2.17 [1.07–4.40]	9 (941)	18 (6708)	0.004*	3.58 [1.60–7.98]
Project Mine	19 (4366)	2 (1832)	0.053	4.00 [0.93–17.15]	17 (4366)	2 (1832)	0.079	3.57 [0.83–15.47]

*AC*, Allele count; *CI*, confidence interval; *OR*, odd ratio. *P < 0.05

### Variants in *KIF1A* associated with ALS are distinct from variants associated with SPG30/HSAN2/MRD9

Mutations in *KIF1A* have been identified to cause SPG30, HSAN2, and MRD9. Considering the shared clinical manifestations between these diseases and ALS, the ALS patients with variants in *KIF1A* were carefully examined to rule out a misdiagnosis. Furthermore, the variants identified in our ALS cohort have not been reported in SPG30, HSAN2, or MRD9, except for the variant p.R370C, which has been reported in a patient with complex SPG [42]. The phenomenon of different phenotypes caused by the same variant in the *KIF1A* gene has also been described previously [27].

**Fig. 1:**
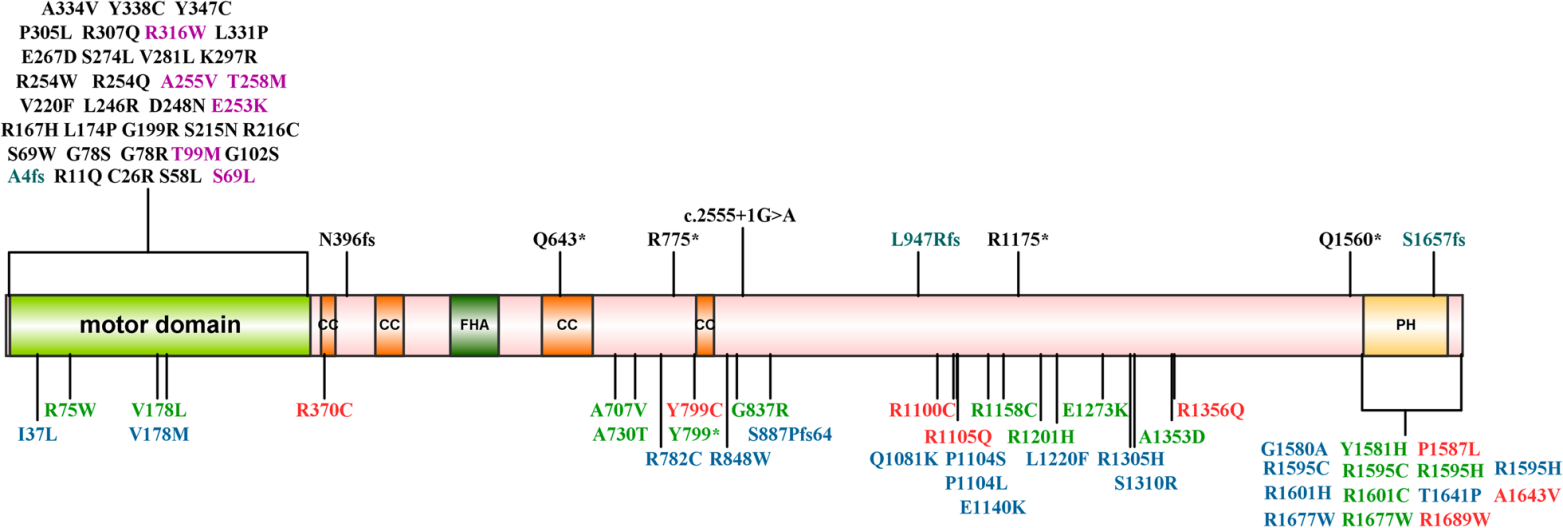
Schematic representation of the KIF1A protein with variants identified in *KIF1A*-related disorders. Variants identified in our cohorts are depicted below the protein schematic: red indicates variants identified in our ALS cohort; blue indicates variants in Project Mine; green indicates variants in ALSdb database. Previously reported variants are listed above the schematic, which are associated with SPG30 (including cases described as MRD9) (black), HSAN2 (blue), and multiple phenotypes (SPG30 and HSAN2) (purple). Details on the patients and variants are shown in Additional file 1: Table S2. Protein domains were determined according to UniProt (https://www.uniprot.org). Variants were annotated with reference to the canonical transcript NM_004321 (p.P886fs was only identified in transcript NM_001244008). Motor domain (amino acids 5–354); CC: coiled coil domains, CC1 (amino acids 366–383), CC2 (amino acids 429–462), CC3 (amino acids 622–681), CC4 (amino acids 801–822); FHA: Forkhead associated domain, amino acids 516–572; PH: pleckstrin homology domain, amino acids 1575–1673

To further elucidate the relationship between *KIF1A* genotype and phenotype, we evaluated the locations of the ALS-related RDVs discovered in our study and pathogenetic variants related with other phenotypes (SPG30, HSAN2, and MRD9) that have been reported in ClinVar (https://www.ncbi.nlm.nih.gov/clinvar/) (Fig. 1). Intriguingly, the variants associated with SPG and HSAN were mostly located in the N-terminal motor domain of KIF1A, while the variants identified with the ALS phenotype in our study were located predominantly in the C-terminal cargo-binding region.

### Clinical characteristics of ALS patients with RDVs in *KIF1A*

The clinical manifestations of the 10 ALS patients carrying RDVs in *KIF1A* varied greatly. The mean AAO of the 10 patients was 56.70 ± 7.82 years, ranging from 40 to 67 years. The clinical features of patients with variants in different functional regions of KIF1A are summarized in Table 3 and Additional file 1: Table S4. Interestingly, we found that these patients tended to display sensory disturbance (60.0%, 6/10).

**Table 3:** Clinical features of ALS patients with RDVs in the *KIF1A* gene

Patient ID	Sample ID	Amino acid change	Sex	Family history	Age at onset (years)	Survival time (months)	Alive (Y/N)	Site of onset	Cognition impairment	Sensory
RDV location (from CC1 to CC2)										
P1	A148	R370C	M	S	59	NA	NA	Spinal	NA	−
RDV location (between CC3 and PH)										
P2	A981	Y799C	M	S	56	50	N	Spinal	+	−
P3	A125	R1100C	F	S	66	28	N	Spinal	NA	+
P4	A707	R1105Q	M	AD	40	> 84	Y	Spinal	−	+
P5	A054	R1356Q	F	S	63	> 42	Y	Spinal	−	+
RDV location (PH domain)										
P6	A737	P1587L	M	S	59	> 56	Y	Spinal	+	+
P7	A342	P1587L	M	S	50	> 41	Y	Spinal	−	+
P8	A893	P1587L	M	S	67	29	N	Bulbar	−	+
P9	A860	A1643V	M	AD	50	32	N	Spinal	−	−
P10	A331	R1689W	M	S	57	> 9	Y	Spinal	−	−

*AD*, autosomal dominant; *F*, female; *M*, male; *N*, no; *NA*, not available; *S*, sporadic; *Y*, yes; +, affected; −, not affected

**Fig. 2:**
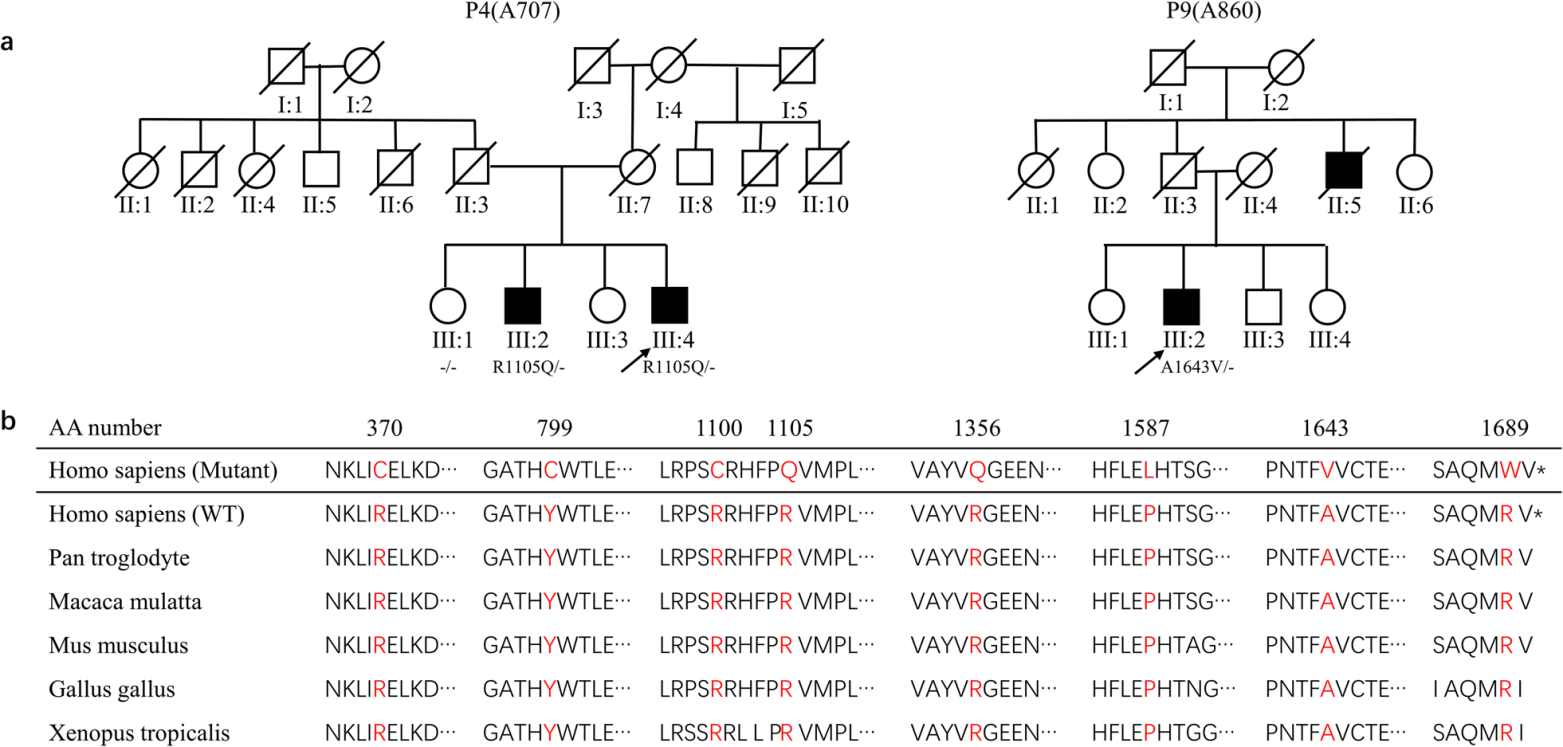
Pedigrees of two FALS patients and evolutionary conservation of mutated KIF1A amino acids. **a** Pedigrees of patients P4 and P9. Arrow, proband; square, man; circle, woman; slashed symbol, deceased; filled symbol, affected; empty symbol, unaffected. **b** Evolutionary conservation of altered amino acids of KIF1A associated with ALS. AA number, amino acid number of human KIF1A protein

Among these patients, two had a family history of ALS (Fig. 2). In Family 1, proband P4 (A707, III-4) harbored the variant c.3314G > A;p.R1105Q and showed early disease onset and long disease duration. His elder brother (III:2) carried the same variant and showed similar clinical features. The elder sister (III:1) did not harbor this variant and did not show neurological phenotypes at her last examination, at the age of 64. In Family 2, Patient P9 (A860) (III:2) harboring the variant p.A1643V showed a short survival time with an AAO of 50 years. His uncle (II:5) showed similar symptoms and died at age 38. The patient’s father did not show neurological symptoms or signs at the latest examination. Unfortunately, because DNA samples from the proband’s father and uncle were unavailable, we did not perform co-segregation analysis on this family.

The other eight patients all had sporadic ALS. Patients P6 (A737), P7 (A342) and P8 (A893) carried the same variant (p.P1587L) in *KIF1A* but showed different disease manifestations. The patient P6 (A737) was diagnosed with probable ALS 34 months after onset at the age of 59, and his motor symptoms were accompanied by cognitive impairment and sensory disturbance. The patient P7 (A342) showed similar manifestations. He was diagnosed with probable ALS 41 months after onset at the age of 53, and he also showed sensory disturbance. The patient P8 (A893) showed a more severe phenotype, with a survival time of only 29 months; he also showed sensory disturbance but without cognitive impairment.

### ALS-associated missense variants of *KIF1A* enhance binding to cargos

**Fig. 3:**
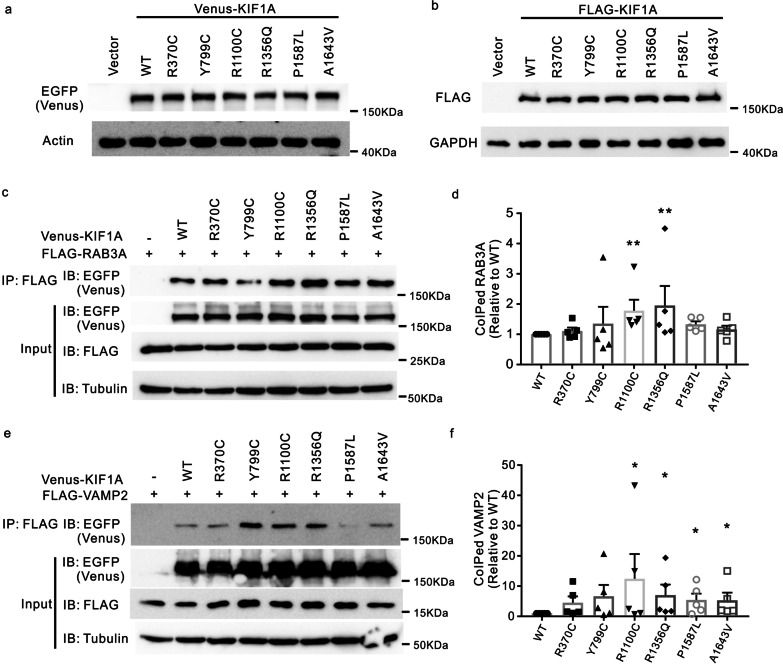
Binding of cargos to ALS-associated KIF1A mutants. **a**, **b** Plasmids expressing Venus- or FLAG-tagged WT and missense-mutation KIF1A were transfected into HEK293T cells. Cells were lysed and immunoblotted with antibodies against EGFP (to recognize Venus) or FLAG. **c**, **e** HEK293T cells were cotransfected with plasmids encoding FLAG-RAB3A or FLAG-VAMP2, along with plasmids encoding Venus-KIF1A WT, designated mutants, or empty vector. FLAG-RAB3A or FLAG-VAMP2 was immunoprecipitated with a FLAG antibody, and the immunoprecipitated proteins were identified by blotting with the indicated antibodies. **d** Quantification of immunoprecipitates in **c**. The intensity of coimmunoprecipitated RAB3A was normalized to the intensity of RAB3A input, and then the value for each mutant was normalized to WT. *n* = 5 experiments. **P < 0.01. *P* = 0.0054 (WT vs R1100C); *P* = 0.0084 (WT vs R1356Q); *P* = 0.0570 (WT vs P1587L); *P* = 0.3055 (WT vs A1643V) by one-way ANOVA and Dunn’s test. Error bars represent SEM. **f** Quantification of immunoprecipitates in **e**. Quantification was performed as described in **d**. *n* = 5 experiments. *P < 0.05. *P* = 0.0281 (WT vs R1100C); *P* = 0.0192 (WT vs R1356Q); *P* = 0.0281 (WT vs P1587L); *P* = 0.0281 (WT vs A1643V) by one-way ANOVA and Dunn’s test. Error bars represent SEM

Next, we investigated the functional effects of several ALS-associated variants, among which Y799C, R1100C, R1356Q, P1587L, and A1643V are located in the C-terminal half of the protein, spanning from the CC3 to the PH domain, while R370C is located in CC1. To test whether these variants affect the protein level of KIF1A, we generated constructs expressing wild-type (WT) or mutated form of human KIF1A and transfected these constructs into HEK293T cells. Immunoblotting showed comparable levels of the mutated KIF1A proteins to that of WT KIF1A (Fig. 3a, b), suggesting that these ALS-associated *KIF1A* variants do not alter protein abundance.

Given that the ALS-related *KIF1A* variants were located predominantly at the C-terminal region, which preferably binds cargos, we sought to test whether these variants alter the binding affinity to cargo proteins. KIF1A is the primary kinesin motor that transports SVPs carrying synaptic vesicle proteins such as RAB3A, synaptophysin, and VAMP2 [20–22]. We expressed WT and mutant KIF1A tagged with Venus (a YFP variant) at the C-terminal together with FLAG-tagged RAB3A or FLAG-tagged VAMP2 in HEK293T cells and evaluated their coimmunoprecipitation with RAB3A or VAMP2. Strikingly, some *KIF1A* missense variants (including R1100C and R1356Q) showed increased binding to RAB3A (Fig. 3c, d, Additional file 1: Fig. S2). In addition, four KIF1A mutants (R1100C, R1356Q, P1587, and A1643V) displayed higher binding affinity to VAMP2 than WT KIF1A (Fig. 3e, f, Additional file 1: Fig. S3). Moreover, we assessed the binding of missense variants of KIF1A to synaptophysin by using coimmunoprecipitation in HEK293T cells. The binding affinity of KIF1A variant Y799C to synaptophysin was enhanced compared to that of WT (Additional file 1: Fig. S4).

### ALS-related *KIF1A* variants increase colocalization of KIF1A motor with SVPs in cultured neurons

**Fig. 4:**
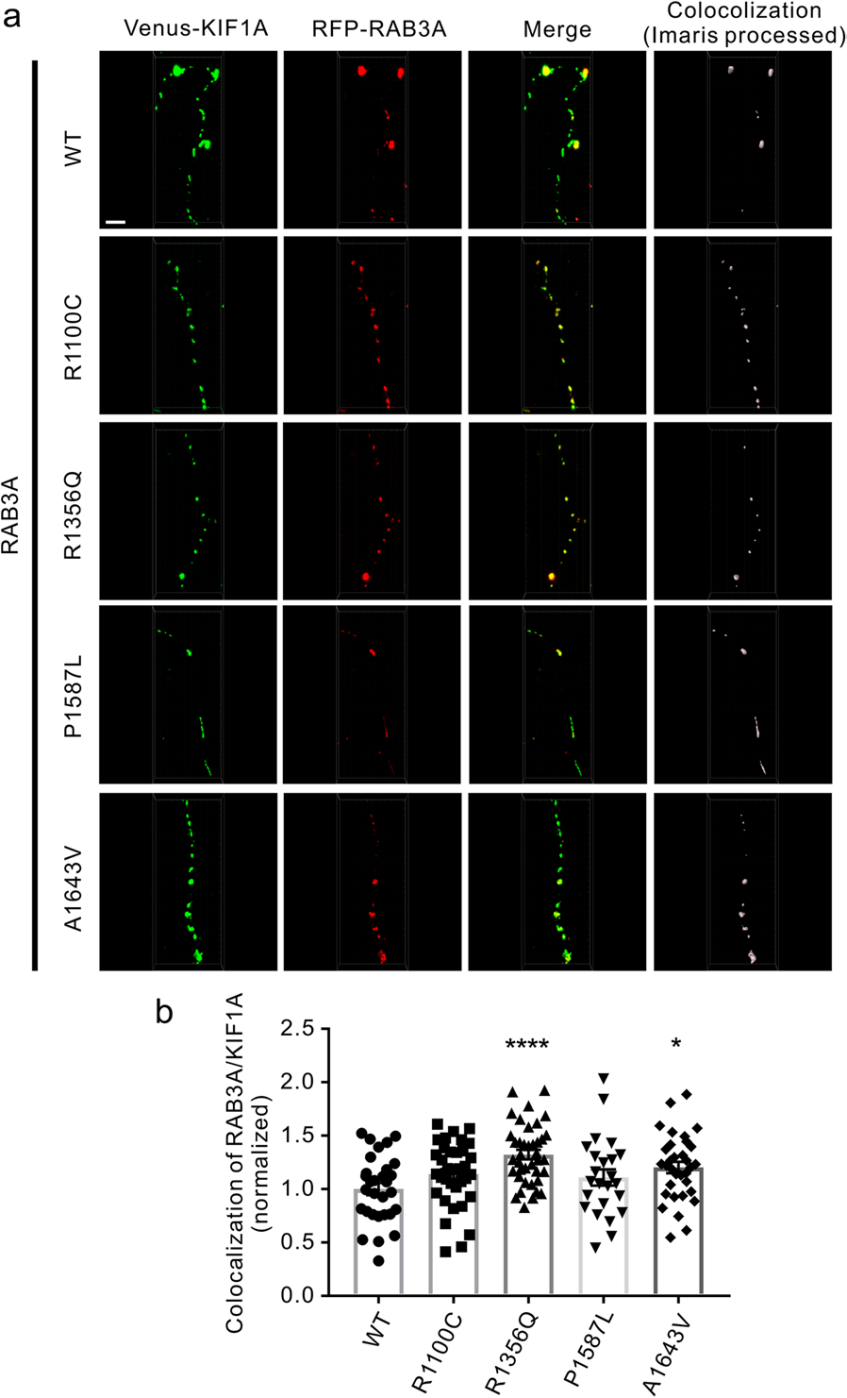
Colocalization of RAB3A with KIF1A variants that carry ALS-associated mutations. **a** Cultured mouse cortical neurons (DIV7) were cotransfected with the RFP-RAB3A construct, along with the Venus-tagged WT and mutant KIF1A constructs. Neurons were fixed and imaged at DIV11. Scale bar, 5 μm. **b** Quantitative analysis of the percentage of RAB3A colocalized with KIF1A in **a**. *n* = 24–39 neurons from three independent experiments. *P < 0.05, ****P < 0.0001; *P* = 0.0659 (WT *vs* R1100C); *P* < 0.0001 (WT *vs* R1356Q); *P* = 0.201 (WT *vs* P1587L); *P* = 0.0104 (WT *vs* A1643V) by one-way ANOVA and Fisher’s test. Error bars represent SEM

We next asked whether the increased cargo-binding capacity of ALS-related *KIF1A* variants is associated with any change in the localization of presynaptic vesicles to the KIF1A motor. We cotransfected the RFP-RAB3A construct along with Venus-tagged mutant (R1100C, R1356Q, P1587L, and A1643V) or WT *KIF1A* into the cultured cortical neurons at DIV7 and analyzed the colocalization of SVPs (containing RAB3A) and KIF1A at DIV11. The results showed that the percentage of RAB3A colocalized with KIF1A was increased in neurons transfected with plasmids expressing the R1356Q and A1643V *KIF1A* mutants (Fig. 4a, b). The R1100C variant also increased the colocalization between RAB3A and KIF1A, although this increase did not reach statistical significance (Fig. 4a, b, *P* = 0.0659).

**Fig. 5:**
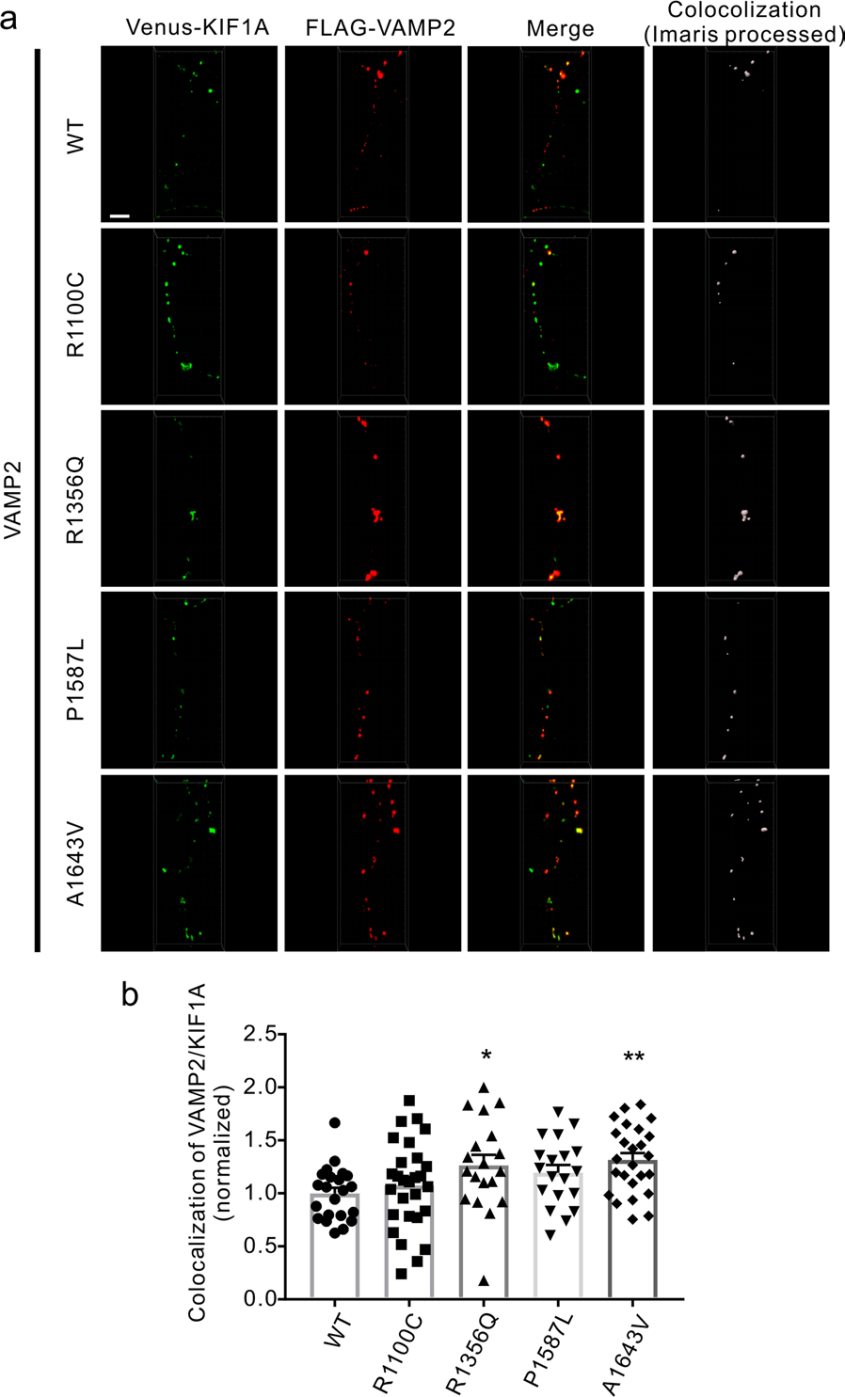
Colocalization of VAMP2 with ALS-associated KIF1A mutants. **a** Cultured mouse cortical neurons (DIV7) were cotransfected with the FLAG-VAMP2 construct, along with the Venus-tagged WT and mutant KIF1A constructs. Neurons were fixed, immunostained with an anti-FLAG antibody, and imaged at DIV11. Scale bar, 5 μm. **b** Quantitative analysis of the percentage of VAMP2 colocalized with KIF1A in **a**. *n* = 19–28 neurons from three independent experiments. *P < 0.05; **P < 0.01; *P* = 0.0196 (WT vs R1356Q); *P* = 0.0030 (WT vs A1643V) by one-way ANOVA and Fisher’s test. Error bars represent SEM

We performed a similar examination of the colocalization of SVPs carrying VAMP2 with the KIF1A motor. The R1356Q and A1643V variants increased the percentage of VAMP2 colocalized with KIF1A (Fig. 5a, b). Together, these data show that presynaptic vesicle precursors accumulate to ALS-associated mutated KIF1A motors.

## Discussion

Here, we provide clinical, genetic, and cell biological data to define a new *KIF1A*-associated phenotype of ALS. We identified eight variants in the *KIF1A* gene from 10 of 941 patients with ALS. The frequency of candidate RDVs in *KIF1A* suggested that *KIF1A* might be a common causative or risk-conferring gene for ALS, especially when the variants are located in the C-terminal half of the protein, a cargo-binding region. However, the significant association of *KIF1A* gene with ALS was not replicated in the Project Mine ALS cohort. This may be caused by difference in genetic background, which is well recognized in ALS. For example, the expansion mutation in *C9orf72* is the most common genetic cause of ALS in Europe, but not in Asia. In addition, mutations in *KIF5A*, identified as a novel ALS gene in a European ancestry genome-wide analysis study, were rare in our ALS patients [2, 29].

In the present study, the ALS patients with RDVs in the *KIF1A* gene showed high clinical heterogeneity. The AAO ranged from 40 to 67 years, and the survival time ranged from more than 9 to more than 84 months. We noted several interesting genotype–phenotype correlation phenomena. For example, a same variant, R370C [42] or P1587L, is associated with different clinical manifestations. We speculate that the different phenotypes in these patients with P1587L might be caused by the difference in disease duration. That is, it is possible that patient P6 might show cognitive impairment with a longer survival time. Interestingly, we found that ALS patients carrying RDVs in *KIF1A* tended to show sensory disturbance. Consistent with this observation, mutations in *KIF1A* have also been reported to cause HSAN, which affects sensory neurons. Etiologically, this could be explained by the various biological functions of the KIF1A protein. KIF1A is required for transportation of the neurotrophin receptor TrKA [24]. TrkA protein is implicated in the sensory pathway [43], and disruption of KIF1A-mediated axon transport of TrKA leads to sensory neuron loss [23]. Therefore, we speculate that the ALS-related variants in *KIF1A* might have diverse functional impacts that result in sensory disturbance in ALS patients.

Notably, we also identified one patient who carried RDV in other ALS pathogenic genes. In addition to p.R370C in *KIF1A*, patient P1 (A148) also carried a c.C312G; p.Y104X variant in the *CHCHD10* gene. This patient showed upper limb and neck weakness and atrophy six months after onset. More detailed clinical information was not available due to the loss of follow-up. The frequency of the c.C312G; p.Y104X variant in the public databases was less than 0.01% and functional prediction suggested pathogenic or uncertain significance in ClinVar. In this patient, the RDV in *KIF1A* and RDVs in other causative genes may both contribute to the pathogenesis of ALS. These results are in line with the oligogenic genetic model of ALS.

Our present results, along with other findings, suggest that *KIF1A* might be a shared causative gene for SPG, HSAN, and ALS. Some other common causative genes for these different types of disease have been reported. For example, pathogenic mutations in either *KIF5A* or *SPG11* lead to SPG and ALS [28–31], and mutations in the *SPTLC1* gene lead to HSAN and ALS [32, 33, 44]. Interestingly, the *KIF1A*-associated HSAN and MRD phenotypes have also been recognized to be mild phenotypes of complex SPG [27]. Therefore, we hypothesize that HSAN, SPG, and ALS may represent a continuum of phenotypes associated with variants in the *KIF1A* gene. As is the case for *NOTCH2NLC, C9ORF72,* and *PRRT2* genes, the clinical spectrum of *KIF1A*-related disease varies greatly [37, 45, 46], giving rise to a large number of “*KIF1A*-related disorders”. The high clinical heterogeneity of “*KIF1A*-related disorders” may be explained by multiple etiological mechanisms, such as the multiple biological functions of the KIF1A protein, potential modifying genes, and epigenetic and environmental factors.

Consistent with previous studies of KIF5A [29, 47], we found that most variants within the KIF1A C-terminal cargo-binding domain are related to ALS, while the majority of missense variants at the N-terminal motor domain are related to SPG. The variety of clinical phenotypes may be caused by the different changes in function caused by variants in different regions of KIF1A. Our results highlight the importance of the cargo-binding activity of the kinesin motor in ALS pathogenesis. Precise regulation of motor-cargo interactions is critical for cargo recruitment, transport efficiency and specificity, and thus for modulating the temporal and spatial distribution of the cargos. Thus far, it is still unclear how cargo recognition, binding, transport, and detachment affect ALS symptoms. In this study, the biochemical and cellular biological examinations revealed that the ALS-related *KIF1A* variants modulate motor-cargo interactions and alter the colocalization of SVPs with the KIF1A motor in neurons. These findings suggest that the disease-associated variants indeed regulate the physiological role of kinesin motors. As KIF1A transports many types of cargo, including SVPs, DCVs, and lysosomes, alterations in the interaction with any cargo might contribute to ALS symptoms. Thus, further comprehensive investigations into the binding and transportation of a wide range of cargos are needed to dissect the pathogenic mechanisms of ALS-associated variants.

In this study, our results revealed that the ALS-related *KIF1A* variants might promote binding to cargos, implicating a gain of function for *KIF1A* variants. Previous results have identified relationships between diseases and gain-of-function mutations in genes involved in axonal transport, such as SPG30 and *KIF1A*, congenital fibrosis of the extraocular muscle type 1 and *KIF21A*, and autosomal dominant lower extremity-predominant spinal muscular atrophy-2A and *BICD2* [48–51]. In addition, a missense mutation in the *Dync1h1* (cytoplasmic dynein heavy chain 1) gene causes progressive motor neuron degeneration in mice [52] and enhances binding to the dynein light intermediate and intermediate chains [53]. We show here that the ALS-related *KIF1A* variants located at the C-terminal increase SVP binding and recruit more SVPs, suggesting that these variants are gain-of-function rather than loss-of-function mutations. Together, these studies indicate that the balance of tightly regulated axon transport events is essential for neuron physiology and survival. It is likely that the abnormal accumulation of SVPs or other KIF1A cargos caused by ALS-related *KIF1A* missense variants is deleterious and neurotoxic, eventually resulting in neurodegeneration.

Autoinhibition is a common mechanism for regulation of Kinesin motor activity, such as KIF1A and KIF5A. Recently, ALS-related mutations in KIF5A have been reported to result in a lack of autoinhibition, leading to a toxic gain of function [54]. KIF5A is autoinhibited by a direct intramolecular interaction between its C-terminal isoleucine-alanine-lysine (IAK) motif and the N-terminal motor domain. ALS-related mutation within the KIF5A C-terminal region disrupts the autoinhibition and leads to a constitutively activated motor. In contrast, current studies have suggested that the CC2-FHA-CC3 region serve as a regulatory region of KIF1A to modulate autoinhibition and dimerization of molecular motor [55–57]. As most of the ALS-associated *KIF1A* variants identified in this study locate at the C-terminal CC4-PH region that is supposed to bind cargo, we did not test the effect of these mutations on autoinhibition. However, it is possible that further genetic studies may identify more ALS-related mutations that regulate motor autoinhibition. The putative mechanism involving autoinhibition and its relation to ALS pathogenesis needs more investigations.

In this study, we tested the cargo-binding capacity of six ALS-related *KIF1A* point mutations. Results indicated a variability in the effect of these mutations on cargo binding, likely because that these mutations locate at different domains of KIF1A motor. Several studies have dissected the role of different KIF1A domains in cargo binding. For example, Hummel et al. showed that CC4 and PH regions are required for KIF1A-cargo interaction [57]; Stucchi et al. reported that the stalk domain of KIF1A (amino acids 657–1105) interacts with Liprin-a and TANC2 to help DCV trafficking [24]. However, the exact binding regions for different cargos remain largely unclear. We found that R1100C and R1356Q, which locate between regions CC4 and PH, alter the interaction with RAB3A and VAMP2. In addition, P1587L and A1643V that locate at the PH domain also alter the binding to VAMP2, while moderately affecting binding to RAB3A (*P* = 0.0570, P1587L; *P* = 0.3055, A1643V). These findings support the notion that CC4 and PH, as well as the region between them, interact with cargos. Moreover, we also noted that several variants, such as R370C, are located in the CC1 region. It has been reported that the CC1 domain does not bind to cargos but regulates dimerization [58, 59]. Consistently, we found that the R370C variant did not exhibit altered binding to SVP cargo proteins in co-immunoprecipitation experiments. The R370C variant might contribute to ALS pathogenesis through a mechanism distinct from that of other variants.

Our study had certain limitations. First, principal component analysis was not performed on the entire cohort to demonstrate the similar ethnic background between patients and controls. Second, the size of the cohort was relatively small compared to other studies of genome-wide variants in ALS. In addition, the *P*-values identified in this study would not reach statistical significance if in a genome-wide study. However, burden analysis in genome-wide studies did not always identify all known ALS causative or risk genes as ALS-associated genes. In a word, it requires more studies to validate the association of *KIF1A* with ALS. Third, due to the late onset of ALS, many family members of the patients in our study were unavailable for co-segregation analysis. Therefore, we were not able to determine whether these variants were de novo variants. Fourth, the sensory disturbance was evaluated only based on subjective description, and quantitative sensory tests were not performed; thus, this relationship of variants in *KIF1A* and sensory disturbance should be verified in the future. Last, in the present study, we conducted only a preliminary functional assay of cargo binding in cultured cells. Careful examination of whether these ALS-related *KIF1A* variants lead to neurodegenerative conditions and neuron loss in animal models will help to further clarify the pathogenic consequences and their relevance to ALS.

## Conclusions

In summary, our study highlights the importance of KIF1A motor-mediated transport in the pathogenesis of ALS, indicating *KIF1A* as an important player in the oligogenic scenario of ALS.

## Supplementary Information

**Additional** **file** **1:** **Fig. S1**. Schematic representation of the KIF1A protein with the RDVs identified in controls. **Fig. S2**. Interaction between RAB3A and the KIF1A motor. **Fig. S3**. Interaction between VAMP2 and the KIF1A motor. **Fig. S4**. ALS-associated KIF1A variants alter binding to synaptophysin. **Table S1**. Primers in constructing plasmid. **Table S2**. Details of RDVS in the KIF1A gene identified in ALS patients and healthy controls. **Table S3**. In-silico pathogenicity predictions for RDVs in the KIF1A gene. **Table S4**. Clinical features in ALS patients carrying RDVs in the KIF1A gene.

## Data Availability

Data analyzed in this study are available from the corresponding authors upon reasonable request. In addition, the genetic variation data have been submitted to dbSNP.

## Abbreviations

AAO: Age at onset
ALS: Amyotrophic lateral sclerosis
CC: Coiled coil
DCV: Dense core vesicle
FALS: Familial ALS
FHA: Forkhead associated domain
HSAN: Hereditary sensory and autonomic neuropathy type
MAF: Minor allele frequency
MRD: Mental retardation type
PH: Pleckstrin homology domain
RDVs: Rare damage variants
SALS: Sporadic ALS
SPG: Spastic paraplegia
SVPs: Presynaptic vesicle precursors
WES: Whole-exome sequencing
