# Supplementary material for: Association of variants in the KIF1A gene with amyotrophic lateral sclerosis
Source: Transl Neurodegener. 2022 Oct 26;11:46. doi: 10.1186/s40035-022-00320-2 (PMC9597953; doi:10.1186/s40035-022-00320-2)
Supplement: Supplementary file 1 — Additional file 1: Fig. S1. Schematic representation of the KIF1A protein with the RDVs identified in controls. Fig. S2. Interaction between RAB3A and the KIF1A motor. Fig. S3. Interaction between VAMP2 and the KIF1A motor. Fig. S4. ALS-associated KIF1A variants alter binding to synaptophysin. Table S1. Primers in constructing plasmid. Table S2. Details of RDVS in the KIF1A gene identified in ALS patients and healthy controls. Table S3. In-silico pathogenicity predictions for RDVs in the KIF1A gene. Table S4. Clinical features in ALS patients carrying RDVs in the KIF1A gene. [file 40035_2022_320_MOESM1_ESM.pdf]

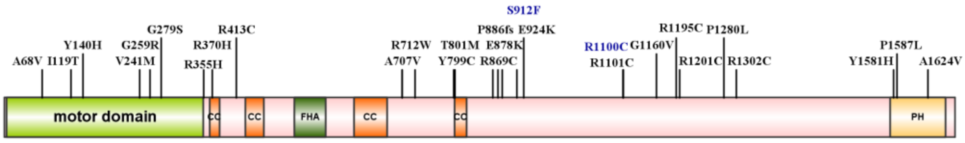

Figure S1 Schematic representation of the KIF1A protein with the RDVs identified in controls. Variants identified in controls are depicted above the protein schematic: black indicates variants identified in individuals without neurological disease of East Asian population in the gnomAD database, and blue indicates variants identified in controls of Project Mine. Variants were annotated with reference to the canonical transcript NM_004321 (p.P886fs was only identified in transcript NM_001244008). Motor domain (5-354 aa); CC: coiled coil domains, CC1(366-383 aa), CC2(429-462 aa), CC3(622-681 aa), CC4(801-822 aa); FHA: Forkhead associated domain, 516-572 aa; PH: pleckstrin homology domain, 1575-1673 aa. aa: amino acids.

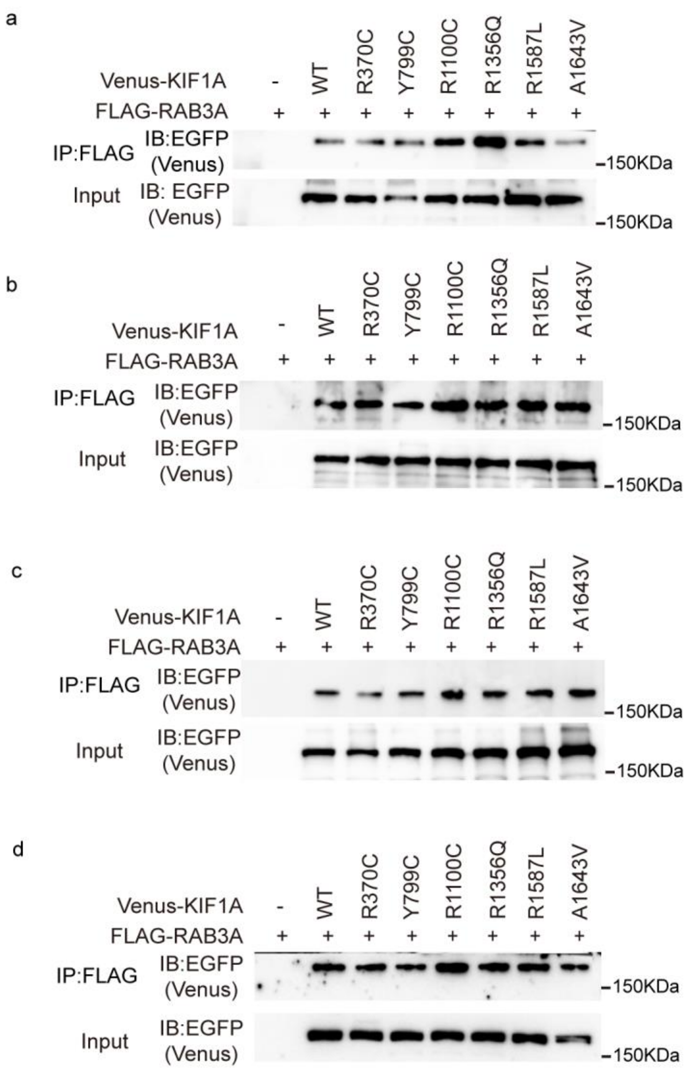

Figure S2 Interaction between RAB3A and the KIF1A motor. (a-d) Coimmunoprecipitation of FLAG-RAB3A with different Venus-tagged WT or mutant KIF1A proteins expressed in HEK293T cells. FLAG-RAB3A was immunoprecipitated with a FLAG antibody and probed for EGFP.

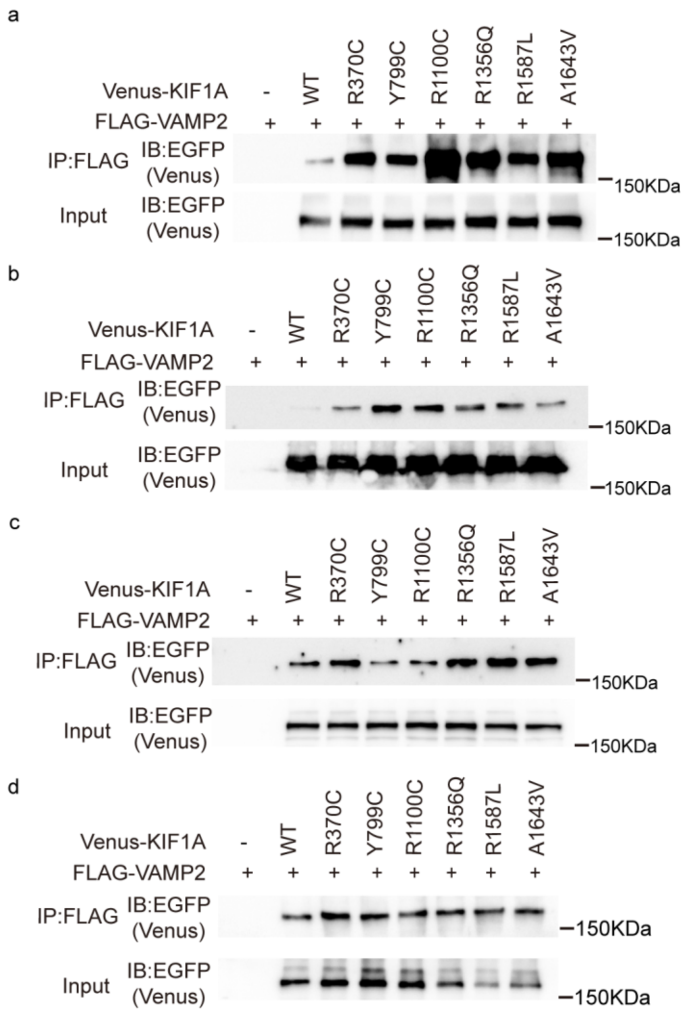

Figure S3 Interaction between VAMP2 and the KIF1A motor. (a-d) Coimmunoprecipitation of FLAG-VAMP2 with different Venus-tagged WT or mutant KIF1A proteins expressed in HEK293T cells. FLAG-VAMP2 was immunoprecipitated with a FLAG antibody, and the immunoprecipitants were probed for EGFP.

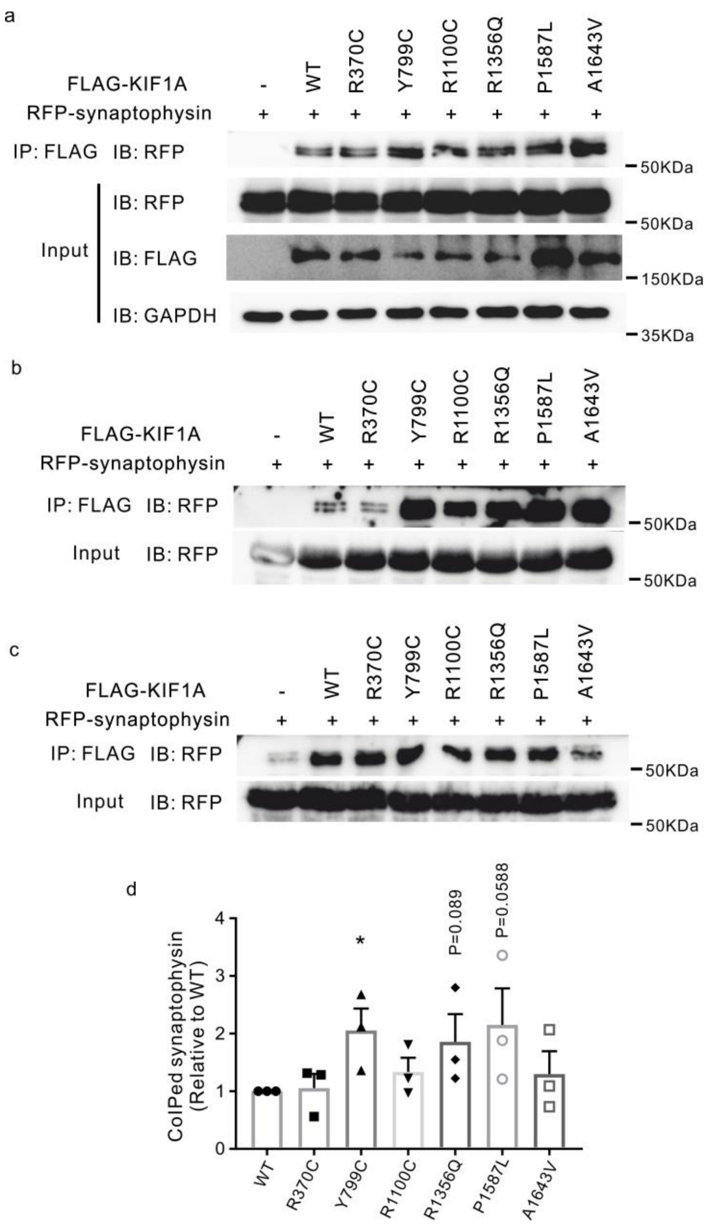

Figure S4 ALS-associated KIF1A variants alter binding to synaptophysin. (a-c) HEK293T cells were cotransfected with an RFP-synaptophysin construct, along with a FLAG-tagged WT or mutant KIF1A construct. FLAG-KIF1A was immunoprecipitated with a FLAG antibody, and the immunoprecipitates were blotted for RFP. **(d)** Quantification of IPs in A-C. n = 3 experiments. $*p < 0.05$ by one-way ANOVA and Dunn's test. Error bars show the SEM.

Supplementary Table 1 Primers in constructing plasmid

**Primers**	**Sequencing (5’-3’)**
KIF1A-FLAG-fwd	agtcaccgtccttgacacgagccaccATGGCCGGGGCTTCGGTG
KIF1A-FLAG-rev	atgggtgattatgatcaatgttacttatcgtcgtcatccttgtaatcgccgctgccGACCCGCATCTGGGCAGAC
KIF1A_fwd	agtcaccgtccttgacacgagccaccATGGCCGGGGCTTCGGTG
KIF1A_rev	tgccgctgccGACCCGCATCTGGGCAGAC
venus_fwd	gatgcgggtcggcagcggcATGGTGAGCAAGGGCGAG
venus_rev	atgggtgattatgatcaatgTTACTTGTACAGCTCGTCCATG
mRFP_fwd	agtcaccgtccttgacacgaagcttgccaccATGGCCTCCTCCGAGGACGTC
mRFP_rev	cggaagccatgccgctgccGGCGCCGGTGGAGTGGCG
Rab3a_fwd	cggcagcggcATGGCTTCCGCCACAGAC
Rab3a_rev	atgggtgattatgatcaatgTCAGCAGGCACAATCCTG
Rab3a-EcoR1	CCGGAATTCAATGGCTTCCGCCACAGACT
Rab3a-BamH1	CGCGGATCCTCAGCAGGCACAATCCTGAT

Supplementary Table 2 Details of RDVS in the *KIF1A* gene identified in ALS patients and healthy controls

Sample	Variant_site	NO of heterozygous	Transcript (NM_004321）	Transcript (NM_001244008)	Functional predictions （ReVe)	gnomAD_exome_ALL	gnomAD_exome_ALL_EAS	gnomAD_genome_ALL	gnomAD_genome_EAS	gnom_AD_non_neuro_ALL	gnom_AD_non_neuro_ALL_EAS
A331	chr2-241656789-G-A	1	exon46:c.C5065T:p.R1689W	exon49:c.C5368T:p.R1790W	0.809	2.07E-05	2.00E-04	-	-		-
A860	chr2-241657569-G-A	1	exon46:c.4928C>T:p.A1643V	exon48:c.5231C>T:p.A1744V	0.72	-	-	-	-	-	-
A737/A893/A342	chr2-241658574-G-A	3	exon45:c.4760C>T:p.P1587L	exon47:c.5063C>T:p.P1688L	0.718	2.87E-05	2.00E-04	6.47E-05	6.00E-04	1.97E-05	7.74E-05
A054	chr2-241662924-C-T	1	exon40:c.4067G>A:p.R1356Q	exon42:c.4370G>A:p.R1457Q	0.903	1.30E-05	-	3.23E-05	-	1.49E-05	-
A707	chr2-241682369-C-T	1	exon32:c.3314G>A:p.R1105Q	exon34:c.3617G>A:p.R1206Q	0.275	8.23E-06	-	3.23E-05	-	9.87E-06	-
A125	chr2-241682385-G-A	1	exon32:c.3298C>T:p.R1100C	exon34:c.3601C>T:p.R1201C	0.819	2.90E-05	-	-	-	2.48E-05	-
A981	chr2-241700103-T-C	1	exon24:c.2396A>G:p.Y799C	exon25:c.2423A>G:p.Y808C	0.856	9.80E-06	1.00E-04	-	-	5.93E-06	9.65E-05
A148	chr2-241712603-G-A	1	exon13:c.1108C>T:p.R370C	exon13:c.1108C>T:p.R370C	0.859	4.06E-06	-	-	-	4.85E-06	-
ALSdb	chr2-241657468-G-A	1	exon46:c.5029C>T:p.R1677W	exon48:c.5332C>T:p.R1778W	0.808	3.01E-05	-	9.70E-05	-	2.06E-05	-
ALSdb	chr2-241658533-G-A	1	exon45:c.4801C>T:p.R1601C	exon47:c.5104C>T:p.R1702C	0.879	4.07E-06	-	-	-	-	-
ALSdb	chr2-241658550-C-T	1	exon45:c.4784G>A:p.R1595H	exon47:c.5087G>A:p.R1696H	0.808	3.26E-05	-	6.47E-05	-	4.88E-05	-
ALSdb	chr2-241658551-G-A	1	exon45:c.4783C>T:p.R1595C	exon47:c.5086C>T:p.R1696C	0.88	2.04E-05	-	-	-	2.44E-05	-
ALSdb	chr2-241658593-A-G	1	exon45:c.4741T>C:p.Y1581H	exon47:c.5044T>C:p.Y1682H	0.754	8.33E-06	5.97E-05	-	-	4.99E-06	7.85E-05
ALSdb	chr2-241662933-G-T	1	exon40:c.4058C>A:p.A1353D	exon42:c.4361C>A:p.A1454D	0.759	6.43E-06	-	-	-	-	-
ALSdb	chr2-241666245-C-T	1	exon37:c.3817G>A:p.E1273K	exon39:c.4120G>A:p.E1374K	0.855	1.26E-05	-	-	-	1.51E-05	-
ALSdb	chr2-241676582-C-T	1	exon36:c.3602G>A:p.R1201H	exon38:c.3905G>A:p.R1302H	0.847	4.06E-05	-	3.24E-05	-	3.88E-05	-
ALSdb	chr2-241679756-G-A	1	exon34:c.3472C>T:p.R1158C	exon36:c.3775C>T:p.R1259C	0.714	8.14E-06	-	-	-	9.73E-06	-
ALSdb	chr2-241697823-C-T	1	exon25:c.2509G>A:p.G837R	exon26:c.2536G>A:p.G846R	0.717	1.64E-05	-	3.23E-05	-	1.47E-05	-
ALSdb	chr2-241700102-G-C	1	exon24:c.2397C>G:p.Y799X	exon25:c.2424C>G:p.Y808X	-	4.91E-06	-	-	-	-	-
ALSdb	chr2-241700696-C-T	1	exon23:c.2188G>A:p.A730T	exon24:c.2215G>A:p.A739T	0.808	4.06E-06	-	-	-	-	-
ALSdb	chr2-241700764-G-A	2	exon23:c.2120C>T:p.A707V	exon24:c.2147C>T:p.A716V	0.803	6.95E-05	3.00E-04	3.23E-05	6.00E-04	7.81E-05	2.31E-04
ALSdb	chr2-241725828-C-G	1	exon6:c.532G>C:p.V178L	exon6:c.532G>C:p.V178L	0.787	4.06E-06	-	-	-	-	-
ALSdb	chr2-241727608-G-A	1	exon4:c.223C>T:p.R75W	exon4:c.223C>T:p.R75W	0.768	6.509E-05	-	3.23E-05	-	5.35E-05	-
Case (Project Mine)	chr2-241657468-G-A	2	exon46:c.5029C>T:p.R1677W	exon48:c.5332C>T:p.R1778W	0.808	3.01E-05	-	9.70E-05	0	2.06E-05	-
Case (Project Mine)	chr2-241657576-T-G	1	exon46:c.4921A>C:p.T1641P	exon48:c.5224A>C:p.T1742P	0.792	-	-	-	-	-	-
Case (Project Mine)	chr2-241658532-C-T	1	exon45:c.4802G>A:p.R1601H	exon47:c.5105G>A:p.R1702H	0.848	1.63E-05	-	-	-	1.46E-05	-
Case (Project Mine)	chr2-241658550-C-T	1	exon45:c.4784G>A:p.R1595H	exon47:c.5087G>A:p.R1696H	0.808	3.26E-05	-	6.47E-05	0	4.88E-05	-
Case (Project Mine)	chr2-241658551-G-A	1	exon45:c.4783C>T:p.R1595C	exon47:c.5086C>T:p.R1696C	0.88	2.04E-05	-	-	-	2.44E-05	-
Case (Project Mine)	chr2-241658595-C-G	1	exon45:c.4739G>C:p.G1580A	exon47:c.5042G>C:p.G1681A	0.83	-	-	-	-	-	-

Supplementary Table 2 Details of RDVS in the *KIF1A* gene identified in ALS patients and healthy controls

Sample	Variant_site	NO of heterozygous	Transcript (NM_004321）	Transcript (NM_001244008)	Functional predictions （ReVe)	gnomAD_exome_ALL	gnomAD_exome_ALL_EAS	gnomAD_genome_ALL	gnomAD_genome_EAS	gnom_AD_non_neuro_ALL	gnom_AD_non_neuro_ALL_EAS
Case (Project Mine)	chr2-241664713-T-G	1	exon38:c.3928A>C:p.S1310R	exon40:c.4231A>C:p.S1411R	0.795	-	-	-	-	-	-
Case (Project Mine)	chr2-241664727-C-T	1	exon38:c.3914G>A:p.R1305H	exon40:c.4217G>A:p.R1406H	0.724	2.09E-05	-	-	-	2.5E-05	-
Case (Project Mine)	chr2-241676526-G-A	1	exon36:c.3658C>T:p.L1220F	exon38:c.3961C>T:p.L1321F	0.819	-	-	-	-	-	-
Case (Project Mine)	chr2-241680714-C-T	1	exon33:c.3418G>A:p.E1140K	exon35:c.3721G>A:p.E1241K	0.866	1.78E-05	-	-	-	1.6E-05	-
Case (Project Mine)	chr2-241682372-G-A	1	exon32:c.3311C>T:p.P1104L	exon34:c.3614C>T:p.P1205L	0.786	-	-	3.23E-05	0.0006	-	-
Case (Project Mine)	chr2-241682373-G-A	1	exon32:c.3310C>T:p.P1104S	exon34:c.3613C>T:p.P1205S	0.775	-	-	-	-	-	-
Case (Project Mine)	chr2-241683399-G-T	1	exon31:c.3241C>A:p.Q1081K	exon33:c.3544C>A:p.Q1182K	0.726	-	-	-	-	-	-
Case (Project Mine)	chr2-241697790-G-A	1	exon25:c.2542C>T:p.R848W	exon26:c.2569C>T:p.R857W	0.861	4.11E-06	-	-	-	4.92E-06	-
Case (Project Mine)	chr2-241700155-G-A	1	exon24:c.2344C>T:p.R782C	exon25:c.2371C>T:p.R791C	0.838	8.76E-06	-	-	-	1.06E-05	-
Case (Project Mine)	chr2-241725828-C-T	1	exon6:c.532G>A:p.V178M	exon6:c.532G>A:p.V178M	0.896	4.06E-06	-	-	-	4.86E-06	-
Case (Project Mine)	chr2-241728727-T-G	1	exon3:c.109A>C:p.I37L	exon3:c.109A>C:p.I37L	0.755	-	-	-	-	-	-
Case (Project Mine)	chr2-241696935-AG-A	1		exon27:c.2658delC:p.P886fs	-	6.76E-06	9.28E-05	.	.	8.06E-06	1.21E-04
Control	chr2-241658463-G-A	1	exon45:c.C4871T:p.A1624V	exon47:c.C5174T:p.A1725V	0.822	4.08E-06	5.81E-05	.	.	4.88E-06	7.71E-05
Control	chr2-241658574-G-A	1	exon45:c.C4760T:p.P1587L	exon47:c.C5063T:p.P1688L	0.718	2.87E-05	2.00E-04	6.47E-05	6.00E-04	1.97E-05	7.74E-05
Control	chr2-241658593-A-G	1	exon45:c.T4741C:p.Y1581H	exon47:c.T5044C:p.Y1682H	0.754	8.33E-06	5.97E-05	.	.	4.99E-06	7.85E-05
Control	chr2-241664737-G-A	1	exon38:c.C3904T:p.R1302C	exon40:c.C4207T:p.R1403C	0.907	1.65E-05	5.85E-05	3.23E-05	0.00E+00	1.49E-05	7.76E-05
Control	chr2-241664802-G-A	1	exon38:c.C3839T:p.P1280L	exon40:c.C4142T:p.P1381L	0.859	1.63E-05	5.81E-05	3.24E-05	0.00E+00	1.96E-05	7.71E-05
Control	chr2-241676583-G-A	1	exon36:c.C3601T:p.R1201C	exon38:c.C3904T:p.R1302C	0.907	1.22E-05	5.80E-05	.	.	9.71E-06	7.69E-05
Control	chr2-241679490-G-A	1	exon35:c.C3583T:p.R1195C	exon37:c.C3886T:p.R1296C	0.72	2.95E-05	7.15E-05	6.46E-05	6.00E-04	3.49E-05	9.25E-05
Control	chr2-241679749-C-A	1	exon34:c.G3479T:p.G1160V	exon36:c.G3782T:p.G1261V	0.917	4.07E-06	5.80E-05	.	.	4.87E-06	7.70E-05
Control	chr2-241682382-G-A	2	exon32:c.C3301T:p.R1101C	exon34:c.C3604T:p.R1202C	0.802	2.90E-05	2.00E-04	.	.	1.99E-05	1.56E-04
Control	chr2-241685585-C-T	3	exon28:c.G2770A:p.E924K	exon30:c.G3073A:p.E1025K	0.751	1.82E-05	3.00E-04	.	.	2.11E-05	3.23E-04
Control	chr2-241689888-C-T	1	exon26:c.G2632A:p.E878K	exon28:c.G2935A:p.E979K	0.822	4.06E-06	5.80E-05	.	.	4.86E-06	7.70E-05
Control	chr2-241689915-G-A	1	exon26:c.C2605T:p.R869C	exon28:c.C2908T:p.R970C	0.876	1.22E-05	5.80E-05	6.46E-05	0.00E+00	1.46E-05	7.69E-05
Control	chr2-241696935-AG-A	1		exon27:c.2658delC:p.P886fs	-	6.76E-06	9.28E-05	.	.	8.06E-06	1.21E-04
Control	chr2-241700097-G-A	1	exon24:c.C2402T:p.T801M	exon25:c.C2429T:p.T810M	0.864	2.06E-05	2.00E-04	.	.	1.87E-05	1.03E-04
Control	chr2-241700103-T-C	1	exon24:c.A2396G:p.Y799C	exon25:c.A2423G:p.Y808C	0.856	9.80E-06	1.00E-04	.	.	5.93E-06	9.65E-05
Control	chr2-241700750-G-A	1	exon23:c.C2134T:p.R712W	exon24:c.C2161T:p.R721W	0.839	4.07E-06	5.80E-05	1.00E-04	0.00E+00	4.86E-06	7.70E-05
Control	chr2-241700764-G-A	3	exon23:c.C2120T:p.A707V	exon24:c.C2147T:p.A716V	0.803	6.95E-05	3.00E-04	3.23E-05	6.00E-04	7.81E-05	2.31E-04

Supplementary Table 2 Details of RDVS in the *KIF1A* gene identified in ALS patients and healthy controls

Sample	Variant_site	NO of heterozygous	Transcript (NM_004321）	Transcript (NM_001244008)	Functional predictions （ReVe)	gnomAD_exome_ALL	gnomAD_exome_ALL_EAS	gnomAD_genome_ALL	gnomAD_genome_EAS	gnom_AD_non_neuro_ALL	gnom_AD_non_neuro_ALL_EAS
Control	chr2-241710465-G-A	1	exon14:c.C1237T:p.R413C	exon15:c.C1264T:p.R422C	0.72	2.03E-05	5.80E-05	.	.	2.43E-05	7.70E-05
Control	chr2-241712602-C-T	1	exon13:c.G1109A:p.R370H	exon13:c.G1109A:p.R370H	0.804	4.06E-06	5.80E-05	.	.	4.85E-06	7.68E-05
Control	chr2-241712647-C-T	1	exon13:c.G1064A:p.R355H	exon13:c.G1064A:p.R355H	0.71	2.84E-05	1.00E-04	.	.	3.39E-05	7.67E-05
Control	chr2-241722490-C-T	1	exon9:c.G835A:p.G279S	exon9:c.G835A:p.G279S	0.942	4.07E-06	5.80E-05	.	.	4.87E-06	7.70E-05
Control	chr2-241723179-C-G	1	exon8:c.G775C:p.G259R	exon8:c.G775C:p.G259R	0.83	5.10E-06	7.04E-05	.	.	6.01E-06	9.13E-05
Control	chr2-241723233-C-T	1	exon8:c.G721A:p.V241M	exon8:c.G721A:p.V241M	0.838	4.92E-06	6.76E-05	.	.	5.83E-06	8.87E-05
Control	chr2-241726679-A-G	1	exon5:c.T418C:p.Y140H	exon5:c.T418C:p.Y140H	0.924	4.07E-06	5.80E-05	.	.	4.87E-06	7.70E-05
Control	chr2-241727475-A-G	3	exon4:c.T356C:p.I119T	exon4:c.T356C:p.I119T	0.994	2.00E-04	3.00E-04	.	.	2.69E-04	3.72E-04
Control	chr2-241727628-G-A	1	exon4:c.C203T:p.A68V	exon4:c.C203T:p.A68V	0.897	2.04E-05	5.80E-05	.	.	2.92E-05	7.70E-05
Control (Project Min	chr2-241686678-G-A	1	exon27:c.2735C>T:p.S912F	exon29:c.3038C>T:p.S1013F	0.813	-	-	-	-	-	-
Control (Project Min	chr2-241682385-G-A	1	exon32:c.3298C>T:p.R1100C	exon34:c.3601C>T:p.R1201C	0.819	2.90E-05	-	-	-	2.48E-05	-

Supplementary Table 3 In-silico pathogenicity predictions for RDVs in the *KIF1A* gene

Sample	Variant_site	SIFT	Polyphen2_ HDIV	Polyphen2_ HVAR	Mutation Taster	Mutation Assessor	PROVEAN	VEST3	MetaSVM	MetaLR	M_CAP	CADD	Functional predictions: pathogenic (total)
A331	chr2-241656789-G-A	D(0.001)	P(1.0)	P(0.995)	D(0.924)	L(1.5)	D(-3.45)	D(0.755)	D(0.003)	T(0.382)	D(0.345)	D(28.5)	9(11)
A860	chr2-241657569-G-A	D(0.004)	D(0.985)	P(0.703)	D(1)	M(2.615)	D(-3.06)	D(0.739)	T(-1.029)	T(0.074)	D(0.079)	D(32)	9(11)
A737/A893/A342	chr2-241658574-G-A	D(0.049)	D(1)	D(1)	D(1)	M(2.665)	D(-4.3)	D(0.647)	T(-1.018)	T(0.095)	D(0.255)	D(31)	9(11)
A054	chr2-241662924-C-T	D(0.002)	D(1)	D(0.987)	D(1)	M(2.83)	D(-3.69)	D(0.78)	D(0.486)	D(0.664)	D(0.833)	D(35)	11(11)
A707	chr2-241682369-C-T	T(0.327)	D(1)	D(0.996)	D(0.916)	M(2.08)	T(-0.75)	T(0.142)	T(-0.232)	T(0.452)	D(0.266)	D(23.2)	6(11)
A125	chr2-241682385-G-A	D(0.007)	D(1)	D(0.998)	D(1)	M(2.65)	D(-5.91)	D(0.76)	D(0.11)	D(0.555)	D(0.352)	D(34)	11(11)
A981	chr2-241700103-T-C	D(0.025)	D(0.98)	P(0.851)	D(1)	M(3.03)	D(-7.54)	D(0.718)	D(0.24)	D(0.553)	D(0.818)	D(25.5)	11(11)
A148	chr2-241712603-G-A	D(0)	D(1)	D(1)	D(1)	H(3.88)	D(-7.15)	D(0.735)	D(0.519)	D(0.708)	D(0.737)	D(33)	11(11)
ALSdb	chr2-241657468-G-A	D(0.001)	D(1)	D(0.995)	D(1)	M(2.375)	D(-6.56)	D(0.637)	D(0.201)	D(0.574)	D(0.573)	D(33)	11(11)
ALSdb	chr2-241658533-G-A	D(0)	D(1)	D(1)	D(1)	M(3.07)	D(-7)	D(0.814)	T(-0.866)	T(0.116)	D(0.5)	D(35)	9(11)
ALSdb	chr2-241658550-C-T	T(0.309)	D(1)	D(1)	D(1)	L(0.95)	T(-1.36)	D(0.833)	T(-0.974)	T(0.124)	D(0.132)	D(31)	6(11)
ALSdb	chr2-241658551-G-A	D(0.001)	D(1)	D(1)	D(1)	M(2.82)	D(-5.69)	D(0.877)	T(-0.761)	T(0.17)	D(0.421)	D(34)	9(11)
ALSdb	chr2-241658593-A-G	D(0)	D(1)	D(0.999)	D(1)	M(2.44)	D(-4.04)	D(0.643)	T(-0.847)	T(0.133)	D(0.279)	D(25.9)	9(11)
ALSdb	chr2-241662933-G-T	T(0.148)	D(0.999)	D(0.979)	D(1)	M(2.615)	D(-3.35)	D(0.852)	T(-0.486)	T(0.316)	D(0.238)	D(25.9)	8(11)
ALSdb	chr2-241666245-C-T	T(0.108)	D(0.995)	P(0.819)	D(1)	M(2.045)	D(-3.31)	D(0.831)	T(-0.264)	T(0.437)	D(0.173)	D(24.8)	8(11)
ALSdb	chr2-241676582-C-T	D(0.002)	D(1)	D(1)	D(1)	M(2.815)	D(-4.53)	D(0.702)	D(0.315)	D(0.595)	D(0.738)	D(34)	11(11)
ALSdb	chr2-241679756-G-A	D(0.032)	D(1)	D(0.956)	D(1)	M(2.3)	D(-2.56)	D(0.642)	T(-0.218)	T(0.414)	D(0.31)	D(34)	9(11)
ALSdb	chr2-241697823-C-T	T(0.101)	D(1)	D(0.992)	D(1)	L(1.27)	D(-2.94)	D(0.702)	T(-0.124)	T(0.477)	D(0.234)	D(29.3)	7(11)
ALSdb	chr2-241700102-G-C	-	-	-	-	-	-	-	-	-	-	-	-
ALSdb	chr2-241700696-C-T	D(0.001)	D(0.992)	P(0.816)	D(1)	M(2.955)	D(-3.51)	D(0.614)	D(0.445)	D(0.663)	D(0.794)	D(34)	11(11)
ALSdb	chr2-241700764-G-A	T(0.139)	P(0.821)	B(0.285)	D(1)	L(1.84)	D(-2.96)	D(0.67)	T(-0.223)	T(0.4)	D(0.398)	D(27)	6(11)
ALSdb	chr2-241725828-C-G	D(0)	D(1)	D(0.999)	D(1)	M(2.31)	D(-2.56)	D(0.552)	D(0.482)	D(0.68)	D(0.652)	D(24.4)	11(11)
ALSdb	chr2-241727608-G-A	D(0.016)	D(0.978)	P(0.531)	D(1)	M(2.855)	D(-4.46)	D(0.551)	D(0.46)	D(0.693)	D(0.251)	D(26.6)	11(11)
Case (Project Mine)	chr2-241657468-G-A	0.001(D)	1(D)	0.995(D)	1(D)	2.375(M)	-6.56(D)	0.637(D)	0.201(D)	0.574(D)	0.573(D)	33(D)	11(11)
Case (Project Mine)	chr2-241657576-T-G	0.003(D)	1(D)	0.999(D)	1(D)	2.81(M)	-5.2(D)	0.755(D)	-1.001(T)	0.101(T)	0.317(D)	26.8(D)	9(11)
Case (Project Mine)	chr2-241658532-C-T	0(D)	1(D)	1(D)	1(D)	3.07(M)	-4.28(D)	0.778(D)	-0.866(T)	0.116(T)	0.38(D)	35(D)	9(11)
Case (Project Mine)	chr2-241658550-C-T	0.309(T)	1(D)	1(D)	1(D)	0.95(L)	-1.36(T)	0.833(D)	-0.974(T)	0.124(T)	0.132(D)	31(D)	6(11)
Case (Project Mine)	chr2-241658551-G-A	0.001(D)	1(D)	1(D)	1(D)	2.82(M)	-5.69(D)	0.877(D)	-0.761(T)	0.17(T)	0.421(D)	34(D)	9(11)
Case (Project Mine)	chr2-241658595-C-G	0(D)	1(D)	1(D)	1(D)	3.17(M)	-5.38(D)	0.697(D)	0.442(D)	0.622(D)	0.643(D)	26.2(D)	11(11)

Supplementary Table 3 In-silico pathogenicity predictions for RDVs in the *KIF1A* gene

Sample	Variant_site	SIFT	Polyphen2_ HDIV	Polyphen2_ HVAR	Mutation Taster	Mutation Assessor	PROVEAN	VEST3	MetaSVM	MetaLR	M_CAP	CADD	Functional predictions: pathogenic (total)
Case (Project Mine)	chr2-241664713-T-G	0.12(T)	0.859(P)	0.491(P)	0.994(D)	2.25(M)	-3.19(D)	0.644(D)	-0.304(T)	0.343(T)	0.203(D)	24.4(D)	8(11)
Case (Project Mine)	chr2-241664727-C-T	0.021(D)	1(D)	0.961(D)	1(D)	2.72(M)	-4.14(D)	0.599(D)	0.109(D)	0.51(D)	0.343(D)	34(D)	11(11)
Case (Project Mine)	chr2-241676526-G-A	0.001(D)	1(D)	1(D)	1(D)	3.185(M)	-3.66(D)	0.674(D)	0.606(D)	0.708(D)	0.839(D)	31(D)	11(11)
Case (Project Mine)	chr2-241680714-C-T	0.017(D)	0.988(D)	0.475(P)	1(D)	2.675(M)	-3.56(D)	0.786(D)	-0.144(T)	0.42(T)	0.438(D)	33(D)	9(11)
Case (Project Mine)	chr2-241682372-G-A	0.001(D)	1(D)	0.999(D)	1(D)	2.585(M)	-8.04(D)	0.635(D)	0.46(D)	0.663(D)	0.623(D)	24.9(D)	11(11)
Case (Project Mine)	chr2-241682373-G-A	0.009(D)	1(D)	0.998(D)	1(D)	2.585(M)	-6.5(D)	0.622(D)	0.299(D)	0.629(D)	0.243(D)	22.9(D)	11(11)
Case (Project Mine)	chr2-241683399-G-T	0.004(D)	0.982(D)	0.968(D)	1(D)	2.195(M)	-3.3(D)	0.528(D)	0.106(D)	0.539(D)	0.375(D)	23.7(D)	11(11)
Case (Project Mine)	chr2-241697790-G-A	0.002(D)	1(D)	0.993(D)	1(D)	2.175(M)	-3.86(D)	0.838(D)	0.146(D)	0.553(D)	0.463(D)	35(D)	11(11)
Case (Project Mine)	chr2-241700155-G-A	0.001(D)	0.989(D)	0.748(P)	1(D)	2.455(M)	-6.35(D)	0.78(D)	-0.106(T)	0.458(T)	0.546(D)	32(D)	9(11)
Case (Project Mine)	chr2-241725828-C-T	0(D)	1(D)	1(D)	1(D)	3.9(H)	-2.56(D)	0.79(D)	0.901(D)	0.822(D)	0.725(D)	24.8(D)	11(11)
Case (Project Mine)	chr2-241728727-T-G	0.208(T)	0.231(B)	0.808(P)	1(D)	0.22(N)	-1.69(T)	0.578(D)	0.008(D)	0.6(D)	0.286(D)	23.5(D)	7(11)
Case (Project Mine)	chr2-241696935-AG-A	-	-	-	-	-	-	-	-	-	-	-	-
Control	chr2-241658463-G-A	D(0.01)	D(1)	D(0.999)	D(1)	L(1.77)	D(-3.45)	D(0.622)	D(0.253)	D(0.562)	D(0.65)	D(32)	10(11)
Control	chr2-241658574-G-A	D(0.049)	D(1)	D(1)	D(1)	M(2.665)	D(-4.3)	D(0.647)	T(-1.018)	T(0.095)	D(0.255)	D(31)	9(11)
Control	chr2-241658593-A-G	D(0)	D(1)	D(0.999)	D(1)	M(2.44)	D(-4.04)	D(0.643)	T(-0.847)	T(0.133)	D(0.279)	D(25.9)	9(11)
Control	chr2-241664737-G-A	D(0)	D(1)	D(0.988)	D(1)	M(2.83)	D(-7.34)	D(0.847)	D(0.391)	D(0.618)	D(0.651)	D(34)	11(11)
Control	chr2-241664802-G-A	T(0.061)	D(1)	D(1)	D(1)	M(2.575)	D(-8.37)	D(0.948)	T(-0.426)	T(0.334)	D(0.208)	D(23.1)	8(11)
Control	chr2-241676583-G-A	D(0)	D(1)	D(1)	D(1)	L(1.715)	D(-7.35)	D(0.902)	D(0.049)	T(0.498)	D(0.702)	D(33)	9(11)
Control	chr2-241679490-G-A	T(0.11)	D(1)	D(0.944)	D(1)	L(1.85)	D(-6.25)	D(0.636)	T(-0.246)	T(0.397)	D(0.161)	D(29.7)	7(11)
Control	chr2-241679749-C-A	D(0.017)	D(1)	D(1)	D(1)	M(2.595)	D(-5)	D(0.916)	D(0.158)	D(0.544)	D(0.601)	D(33)	11(11)
Control	chr2-241682382-G-A	D(0.004)	D(1)	D(0.998)	D(1)	M(2.56)	D(-6.26)	D(0.696)	D(0.209)	D(0.574)	D(0.432)	D(34)	11(11)
Control	chr2-241685585-C-T	T(0.327)	D(1)	D(0.992)	D(1)	M(2.105)	T(-1.28)	D(0.733)	T(-0.479)	T(0.388)	D(0.159)	D(28.2)	7(11)
Control	chr2-241689888-C-T	T(0.105)	D(1)	D(0.992)	D(1)	M(2.455)	D(-3.27)	D(0.779)	T(-0.001)	D(0.524)	D(0.467)	D(35)	9(11)
Control	chr2-241689915-G-A	D(0.001)	D(1)	D(0.997)	D(1)	M(2.67)	D(-5.94)	D(0.785)	D(0.343)	D(0.614)	D(0.533)	D(34)	11(11)
Control	chr2-241696935-AG-A	-	-	-	-	-	-	-	-	-	-	-	-
Control	chr2-241700097-G-A	D(0.001)	D(0.99)	P(0.781)	D(1)	M(2.34)	D(-3.45)	D(0.789)	T(-0.082)	T(0.449)	D(0.519)	D(29.3)	9(11)
Control	chr2-241700103-T-C	D(0.025)	D(0.98)	P(0.851)	D(1)	M(3.03)	D(-7.54)	D(0.718)	D(0.24)	D(0.553)	D(0.818)	D(25.5)	11(11)
Control	chr2-241700750-G-A	D(0.006)	D(1)	D(0.978)	D(1)	M(2.695)	D(-6.15)	D(0.786)	D(0.125)	D(0.537)	D(0.565)	D(34)	11(11)
Control	chr2-241700764-G-A	T(0.139)	P(0.821)	B(0.285)	D(1)	L(1.84)	D(-2.96)	D(0.67)	T(-0.223)	T(0.4)	D(0.398)	D(27)	6(11)

Supplementary Table 3 In-silico pathogenicity predictions for RDVs in the *KIF1A* gene

Sample	Variant_site	SIFT	Polyphen2_ HDIV	Polyphen2_ HVAR	Mutation Taster	Mutation Assessor	PROVEAN	VEST3	MetaSVM	MetaLR	M_CAP	CADD	Functional predictions: pathogenic (total)
Control	chr2-241710465-G-A	T(0.061)	D(1)	D(0.984)	D(1)	M(2.34)	D(-3.18)	D(0.612)	D(0.073)	D(0.512)	D(0.272)	D(28.7)	10(11)
Control	chr2-241712602-C-T	D(0)	D(1)	D(1)	D(1)	H(3.88)	D(-4.47)	D(0.663)	D(0.704)	D(0.694)	D(0.748)	D(34)	11(11)
Control	chr2-241712647-C-T	T(0.116)	P(0.839)	B(0.197)	D(1)	M(2.195)	T(-1.94)	D(0.561)	D(0.175)	D(0.564)	D(0.172)	D(24)	8(11)
Control	chr2-241722490-C-T	D(0.001)	D(1)	D(0.984)	D(1)	M(2.85)	D(-4.88)	D(0.845)	D(0.968)	D(0.863)	D(0.755)	D(28.1)	11(11)
Control	chr2-241723179-C-G	D(0.001)	D(0.996)	D(0.92)	D(1)	M(3)	D(-6.53)	D(0.824)	D(0.914)	D(0.833)	D(0.747)	D(27.8)	11(11)
Control	chr2-241723233-C-T	D(0.001)	D(1)	D(0.983)	D(1)	M(2.02)	T(-2.34)	D(0.615)	D(0.755)	D(0.787)	-	D(30)	9(11)
Control	chr2-241726679-A-G	T(0.099)	D(0.999)	D(0.996)	D(1)	M(2.125)	D(-4.14)	D(0.849)	D(0.149)	D(0.571)	D(0.498)	D(23.5)	10(11)
Control	chr2-241727475-A-G	D(0)	D(1)	D(1)	D(1)	M(2.54)	D(-4.27)	D(0.972)	D(0.49)	D(0.66)	D(0.894)	D(26.8)	11(11)
Control	chr2-241727628-G-A	D(0.004)	D(1)	D(1)	D(1)	L(1.77)	D(-2.85)	D(0.78)	D(0.729)	D(0.78)	D(0.638)	D(29.5)	10(11)
Control (Project Mine)	chr2-241682385-G-A	0.007(D)	1(D)	0.998(D)	1(D)	2.65(M)	-5.91(D)	0.76(D)	0.11(D)	0.555(D)	0.352(D)	34(D)	11(11)
Control (Project Mine)	chr2-241686678-G-A	0.052(T)	1(D)	0.979(D)	1(D)	2.75(M)	-3.91(D)	0.69(D)	-0.166(T)	0.515(D)	0.249(D)	25.2(D)	9(11)

Abbreviations: CADD: combined annotation dependent depletion; LR: logistic regression; M-CAP: Mendelian clinically applicable pathogenicity; PolyPhen2 HDIV: polymorphism phenotyping version 2 human diversity; PolyPhen2 HVAR: polymorphism phenotyping version 2 human variation; PROVEAN: Protein Variation Effect Analyzer; SIFT: sorting intolerant from tolerant; SVM: support vector machine; VEST3: Variant Effect Scoring

Notes: SIFT (D: Damaging; T: Tolerable); PolyPhen2 HDIV (D: Probably_Damaging; P: Possibly_Damaging); Polyphen2_HVAR (D: Probably_Damaging; P: Possibly_Damaging; B:Benign); Mutation Taster (D: Disease_causing); Mutation Assessor (H: High; M: Medium; L: Low; N: Neutral); PROVEAN (D: Damaging; T: Tolerable); VEST3(D: Damaging; T: Tolerable); MetaSVM (D: Damaging; T: Tolerable); MetaLR (D: Damaging; T: Tolerable); M_CAP (D: Damaging); CADD: (D: Damaging; T: Tolerable)

Supplementary Table 4 Clinical features in ALS patients carrying RDVs in the *KIF1A* gene

Clinical features	P 1	P 2	P 3	P 4	P 5	P 6	P 7	P 8	P 9	P 10
ID	A148	A981	A125	A707	A054	A737	A342	A893	A860	A331
Variation (Amino acid change)	R370C	Y799C	R1100C	R1105Q	R1356Q	P1587L	P1587L	P1587L	A1643V	R1689W
Sex	M	M	F	M	F	M	M	M	M	M
Family history	S	S	S	AD	S	S	S	S	AD	S
Age at onset (age)	59	56	66	40	63	59	50	67	50	57
Survival time(months)	NA#	50*	28*	> 84	>42	>56	41	29*	32*	9
Site of onset	Spinal	Spinal	Spinal	Spinal	Spinal	Spinal	Spinal	Bulbar	Spinal	Spinal
Weakness	UL, neck	G	G	LL	G	G	G	G	G	G
Atrophy	UL, neck	G	G	LL	G	G	G	G	G	G
Muscle fasciculation	UL, neck	G	-	NA	G	G	G	G	G	G
Dysarthria	-	Mi	Mo	-	Mi	Mi	Mo	Se	Mi	Mi
Dysphagia	-	-	Mi	-	Mo	Mi	Mi	Se	-	-
Respiratory	-	+	+	-	+	+	-	+	+	+
Cognition impairement	NA	Language dysfunction and memory impairment	NA	-	-	Executive dysfunction and memory impairment	-	-	-	-
Autonomic dysfunction	NA	NA	NA	+	+	+	+	NA	NA	NA
Sensory	-	-	+(pain)	+(hypaesthes	+(numb)	+(pain)	+(numb)	+(pain)	-	-
Reflexes	Hyper(UL)	Hypo	Hyper	Hyper	Hyper	Hyper	Hyper	Hyper	Hyper	Hypo
Other UMN signs	-	-	-	Left Barbinski signs(+),left Hoffman(+) ,ankle	-	Pathological crying and laughing, increased muscle tone of LL	Barbinski sign(+),increa sed muscle tone	-	Increased mus	-
Tongue wasted and fascicualtion	-	+	+	-	-	+	-	+	+	-
Jaw jerk	Absent	Present	Absent	Present	Present	Present	Absent	Absent	Absent	Absent
Neurophysiology										
EMG	CR(cervial)	OD and CR	OD and CR	OD and CR (lumber)	OD and CR	OD and CR	OD(cervial)	-	CR(cervial)	OD and CR
Motor nerve conduction	-	Decreased CMAP and MCV of left median nerve	-	-	-	Decreased CMAP of median and ulnar nerve	-	-	-	-
Sensory nerve conduciton	-	-	-	-	-	Decreased SNAP and SCV of median and ulnar nerve	-	Decreased SCV of left median nerve	-	-

Abbrevations: AD: autosomal dominant; CMAP:compound muscle action potential; CR: chronic renervation; F: feamale; G:Global; Hper: hyperreflexia; Hypo:hyporeflexia; LL: lower limbs; M: male; MCV: motor conduction velocity; Mi: Mild; Mo:Moderate; NA: not abailbale; OD:ongoing denervation; S:sporadic; Se:Severe; SCV:sensory conduction velocity; SNAP: sensory nerve action potentia; UL: upper limbs; "#": loss to follow up; "*": The patient was dead at the time of the study;"+":affected;"-": normal .
